# A pan-cancer analysis of CpG Island gene regulation reveals extensive plasticity within Polycomb target genes

**DOI:** 10.1038/s41467-021-22720-0

**Published:** 2021-04-30

**Authors:** Yueyuan Zheng, Guowei Huang, Tiago C. Silva, Qian Yang, Yan-Yi Jiang, H. Phillip Koeffler, De-Chen Lin, Benjamin P. Berman

**Affiliations:** 1 Department of Medicine, Samuel Oschin Comprehensive Cancer Institute, Cedars-Sinai Medical Center, Los Angeles, CA USA; 2 Department of Pathology, Shantou University Medical College, Shantou, Guangdong People’s Republic of China; 3 Center for Bioinformatics and Functional Genomics, Cedars-Sinai Medical Center, Los Angeles, CA USA; 4 Department of Developmental Biology and Cancer Research, Institute for Medical Research Israel-Canada, Hebrew University-Hadassah Medical School, Jerusalem, Israel

**Keywords:** Cancer genomics, Epigenomics

## Abstract

CpG Island promoter genes make up more than half of human genes, and a subset regulated by Polycomb-Repressive Complex 2 (PRC2^+^-CGI) become DNA hypermethylated and silenced in cancer. Here, we perform a systematic analysis of CGI genes across TCGA cancer types, finding that PRC2^+^-CGI genes are frequently prone to transcriptional upregulation as well. These upregulated PRC2^+^-CGI genes control important pathways such as Epithelial-Mesenchymal Transition (EMT) and TNFα-associated inflammatory response, and have greater cancer-type specificity than other CGI genes. Using publicly available chromatin datasets and genetic perturbations, we show that transcription factor binding sites (TFBSs) within distal enhancers underlie transcriptional activation of PRC2^+^-CGI genes, coinciding with loss of the PRC2-associated mark H3K27me3 at the linked promoter. In contrast, PRC2-free CGI genes are predominantly regulated by promoter TFBSs which are common to most cancer types. Surprisingly, a large subset of PRC2^+^-CGI genes that are upregulated in one cancer type are also hypermethylated/silenced in at least one other cancer type, underscoring the high degree of regulatory plasticity of these genes, likely derived from their complex regulatory control during normal development.

## Abstract (web summary)

A subset of CpG Island promoter genes are regulated by Polycomb-Repressive Complex 2 (PRC2^+^-CGI), which become DNA hypermethylated and silenced in cancer. Here, the authors investigate the transcriptomic and epigenomic characteristics of PRC2-occupied CGI and free CGI across pan-cancer types.

## Introduction

Tumorigenesis is a highly complex process driven by both genetic and epigenetic alterations. Among these abnormalities, cancer-specific DNA hypermethylation at CpG-Island (CGI) promoters is perhaps the most well-established epigenetic deregulation. DNA hypermethylation results in transcriptional repression of a large number of genes in cancer. While some are known tumor suppressors, such as *BRCA1*, *MLH1*, and *VHL*, the majority of such hypermethylated genes are “passengers” (little or no functional contribution to cancer biology). In virtually every cancer type, hundreds of CGI promoters are DNA hypermethylated^1^.

CGI promoters make up a large class of promoters in vertebrate genomes (55–75% of all transcription start sites (TSSs))^2^, and only a small fraction are targeted by DNA hypermethylation in cancer. Generally, CGI promoters fall into two major classes: those associated with genes ubiquitously expressed across most cell types (i.e., “housekeeping” genes), and those under complex regulation during embryonic development, which are typically marked with Polycomb group (PcG) proteins^3^. Both of these classes are unmethylated in embryonic stem cells (ESCs) and most other cell types, but the latter class is prone to DNA hypermethylation in cancer^4–6^. In fact, PcG-associated genes account for more than 75% of all DNA hypermethylated CGI promoters^7^. Most of these appear to be passengers, albeit there is a subset with tumor suppressor function, including *SFRP5*^8^, *GATA5*^9^, and *RUNX3*^10^. A subgroup of highly regulated developmental transcription factors (TFs) have much longer (>5 kilobase) regions of de-methylated and CGI-containing DNA^11^, and these “DNA methylation valleys” (DMVs) also gain methylation in cancer^12,13^.

Initially discovered in Drosophila melanogaster, PcG factors play a major role in the regulation of cell fate and differentiation^14^. They form multiple Polycomb-Repressive Complexes (PRCs), including PRC1 and PRC2. Compared with PRC1, the function and regulation of PRC2 is more extensively characterized and better understood^14^. In mammals, PRC2 is ubiquitously expressed and preferentially binds to CGI promoters to mediate mono-, di- and tri-methylation of histone H3 lysine 27 (H3K27me1/me2/me3)^14,15^. Among them, H3K27me3 is considered as a hallmark of PcG-dependent transcriptional repression. The methyltransferase activity of PRC2 is regulated by three core components, enhancer of zeste homolog 1 (EZH1) or EZH2, suppressor of zeste 12 (SUZ12) and embryonic ectoderm development (EED)^15^. Mechanistically, the susceptibility of PcG-occupied CGI promoters to DNA hypermethylation may be related to the capability of EZH2 in recruiting DNMT3A^16^. Functionally, in addition to maintaining transcriptional repression, PRC2 also establishes a unique “bivalent” chromatin in many unmethylated CGI promoters, which is especially prominent in ESCs^17^. Harboring both H3K27me3 and active histone marks (H3K4me2/3), bivalent chromatin is considered to maintain a low but poised transcriptional state either for rapid activation in specific developmental context or long-term repression in other cell types.

While numerous studies have focused on the hypermethylation and epigenetic silencing of PRC2-associated genes, very few have looked systematically at how the entire class of PRC2-occupied CGI promoters are dysregulated in cancer. Isolated studies have indicated that these promoters might not only be prone to silencing, but also to transcriptional activation in cancer^18^. For example, in colon cancer, many stem cell regulators and proliferation-promoting factors with bivalent promoters became active after losing PcG mark H3K27me3^19^. In addition, a few PRC2-regulated genes, such as the well-defined leukemic oncogene *HOXA9*, have been observed to be either DNA hypermethylated or transcriptionally upregulated in different cancer types^20^. *DLX5*, which contains a bivalent promoter in ESCs and most normal tissues, is converted to an active chromatin state in squamous cancers and can promote proliferation and migration in these cancer types (manuscript under review). These observations indicate that although Polycomb-occupied promoters are well-known to become DNA hypermethylated and epigenetically silenced in cancer, the opposite change may also be common in cancer and play a role in tumor biology. However, the prevalence of this type of transcriptional activation (i.e., how many promoters are affected) in the full spectrum of human cancers, the biological significance of the alteration, and the underlying mechanisms of the transcriptional activation are unknown, possibly because this change has little or no effect on promoter DNA methylation.

In this work, we comprehensively analyze the transcriptomic and epigenomic characteristics of PRC2-occupied CGI (referred to as PRC2^+^-CGI) genes and PRC2-free CGI (referred to as PRC2^−^-CGI) genes using tumor and nonmalignant samples across pan-cancer types. In addition to the expected epigenetically silenced genes, we also find a significant subset of PRC2-occupied CGI promoters that is upregulated in cancer. This class of CGI genes shows the highest degree of tissue-specificity and transcriptional plasticity, belongs to important cancer pathways, and is predominantly controlled by distal enhancers.

## Results

### Systematic identification of transcriptionally deregulated PRC2^+^-CGI and PRC2^−^-CGI genes across human cancers

**Fig. 1:**
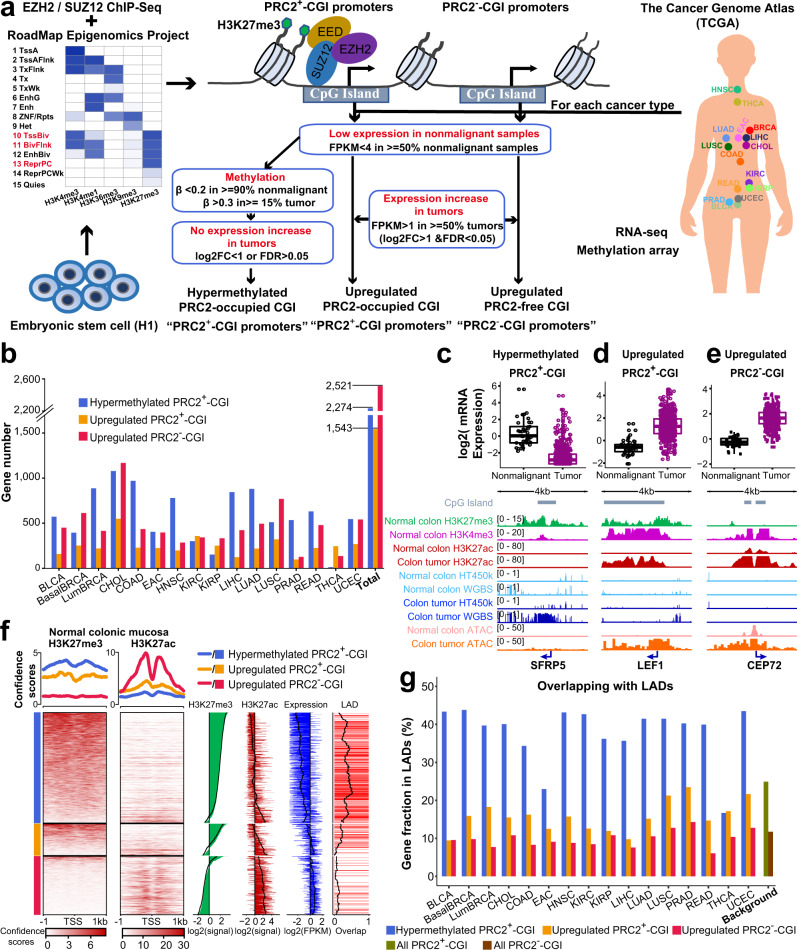
Systematic identification of transcriptionally deregulated PRC2^+^-CGI and PRC2^−^-CGI genes across human cancers. **a** An integrated pipeline for identification of three different classes of CGI promoters. The cancer type information is listed in Supplementary Table 1. **b** The criteria specified in Fig. 1a were applied to each cancer type separately, and the numbers of CGI genes in each class are shown for each TCGA cancer type. **c**–**e** Expression boxplots and Integrative Genomics Viewer (IGV) plots show representative colon adenocarcinoma (COAD) genes for three classes: **c** hypermethylated PRC2^+^-CGI, **d** upregulated PRC2^+^-CGI and **e** upregulated PRC2^−^-CGI. ChIP-Seq data are from Roadmap and ENCODE projects. Expression and methylation data are from TCGA. For **c**–**e**, box plots indicate the median (middle line), 25th, 75th percentile (box) and 5th and 95th percentile (whiskers); *n* = 41 biologically independent nonmalignant samples and 456 colon tumor samples. **f** Three classes of genes identified in COAD show different H3K27me3 and H3K27ac patterns in normal colonic mucosa. The heatmap was ordered by H3K27me3 signal within each class. The barplots show the mean H3K27me3 signal, mean H3K27ac signal, mean expression in normal colonic mucosa and promoter fractions overlapping with lamina-associated domains (LADs). Black lines in the barplots show the trend for a moving window of 100 genes. **g** The fractions of three classes of genes overlapping with LADs across all cancer types.

To characterize alterations in PRC2-occupied CGI promoters in cancer (referred to as PRC2^+^-CGI), we first curated a comprehensive set of TSSs consisting of 101,819 loci from GENCODE and 43,164 from the FANTOM4 Cap-Assisted Gene Expression dataset (Supplementary Fig. 1). A total of 53,860 promoters associated with these TSSs were covered by methylation probes on the HM450K methylation array, and approximately 70% (35,686/53,860) of these promoters overlapped a CGI region. Among CGI promoters, more than 20% (7,573/35,686) were defined as PRC2^+^ in ESCs, based on the annotation of repressed/bivalent ChromHMM states^21^, as well as EZH2/SUZ12-binding (Fig. 1a). We analyzed the same datasets and identified 21,226 CGI promoters without any evidence for PRC2-occupancy (termed PRC2^−^-CGI, Fig. 1a and Methods).

Using ESC chromatin marks to define PRC2^+^ and PRC2^−^ gene classes has been a common practice in the definition of CpG Island Methylator Phenotype (CIMP) and other cancer methylation signatures, due to the more diffuse distribution of H3K27me3 ChIP-Seq in differentiated cell types and the fact that PRC2 ChIP-Seq has been attempted in very few differentiated cell types^6,7,22,23^. This approach is valid because most regions that are PRC2-occupied in ESCs retain H3K27me3 across differentiated cell types of all lineages^12^. Nevertheless, we sought to confirm that these annotations were representative of the PRC2-occupied state in normal tissues. Since PRC2-occupancy is associated with repressed/bivalent transcription, we reasoned that PRC2^+^-CGI genes should have minimal expression in normal tissues. We thus analyzed the mRNA levels of PRC2^+^-CGI genes in TCGA nonmalignant samples which had available histone markers from the NIH Roadmap project (including colonic mucosa, lung, breast epithelium, rectum, esophagus, uterus and liver). As anticipated, H3K27ac signals of PRC2^+^-CGI genes were significantly correlated with their mRNA expression levels, and most were low for both. PRC2^+^-CGI genes with an FPKM greater than 4 showed a marked increase in H3K27ac in most cell types (Supplementary Fig. 2a), and the H3K27me3 mark, a hallmark of PRC2-occupancy, was only positive in PRC2^+^-CGI genes with FPKM < 4 (Supplementary Fig. 2b). As expected, the majority (an average of 77.1%) of PRC2^+^-CGI genes had FPKM < 4 in nonmalignant tissues. These results are consistent with previous reports that a large fraction of silenced H3K27me3-covered regions are shared between ESCs and differentiated cells^12^.

We next cataloged cancer-associated changes in genes associated with CGI promoters by analyzing the transcriptomic and DNA methylation data of TCGA cancer types. A total of 16 TCGA cancer types with sufficient nonmalignant samples (*n* ≥ 5) were used, including bladder cancer (BLCA), basal breast cancer (BasalBRCA), luminal breast cancer (LumBRCA), cholangiocarcinoma (CHOL), colon adenocarcinoma (COAD), esophageal adenocarcinoma (EAC), head and neck squamous cell carcinoma (HNSC), kidney renal clear cell carcinoma (KIRC), kidney renal papillary cell carcinoma (KIRP), liver hepatocellular carcinoma (LIHC), lung adenocarcinoma (LUAD), lung squamous cell carcinoma (LUSC), prostate adenocarcinoma (PRAD), rectum adenocarcinoma (READ), thyroid carcinoma (THCA) and uterine corpus endometrial carcinoma (UCEC) (Supplementary Table 1). As described below, we independently analyzed each of these 16 TCGA cancer types, using transcriptome and DNA methylation profiles to define three distinct gene groups: hypermethylated PRC2^+^-CGI, upregulated PRC2^+^-CGI, and upregulated PRC2^−^-CGI (Fig. 1a and Methods).

A total of 4,378 genes were associated with the 7,573 PRC2^+^-CGI promoters, and we first identified hypermethylated PRC2^+^-CGI promoters (Fig. 1b and Supplementary Table 2) using criteria based on those developed by the TCGA consortium^24^. Consistent with well-established findings^1^, almost 52% of PRC2^+^-CGI genes (2,274 of 4,378) became hypermethylated in at least one cancer type, corresponding to 4,260 promoters (56% of all PRC2^+^-CGI promoters). Most cancer types (12/16) had > 400 hypermethylated PRC2^+^-CGI genes (Fig. 1b), confirming the pervasiveness of this type of epigenetic silencing across human cancers. For the example of COAD, we verified known hypermethylated tumor suppressors such as *SFRP5*^8^, *GATA5*^9^, and *RUNX3*^10^ (Supplementary Data 1). As shown in Fig. 1c, *SFRP5* harbors a bivalent promoter in normal colon tissue, with both H3K27me3 and H3K4me3 signals, and devoid of H3K27ac; in comparison, *SFRP5* becomes DNA hypermethylated, inaccessible (undetectable ATAC-Seq signal) and strongly repressed in colon cancer.

In addition to this hypermethylated group, more than 35% of PRC2^+^-CGI genes (1,543 of 4,378, associated with 2,891 promoters) were upregulated in one or more cancer types (Fig. 1b). On average, each cancer type possessed 245 (ranging from 98 to 549) such upregulated PRC2^+^-CGI genes (Supplementary Table 2). Consistent with a previous study^19^, our analyses in colon cancer revealed many upregulated PRC2^+^-CGI genes associated with the WNT signaling pathway, such as *LEF1*, *LGR5*, *WNT2* (Supplementary Data 2). Shown as an example, the *LEF1* gene is marked with both H3K27me3 and H3K4me3 and devoid of H3K27ac in normal colon tissue. In contrast, it is transcriptionally upregulated in colon tumors and is accompanied by high accessibility, conspicuous H3K27ac levels, and low DNA methylation (Fig. 1d).

For comparison, we characterized upregulation of the PRC2^−^-CGI gene class, which is considered to have relatively ubiquitous expression across different cell types^3^. We applied the identical criteria for upregulation and identified 2,521 upregulated PRC2^−^-CGI genes in one or more cancer types (Supplementary Table 2 and Supplementary Data 3). Most cancer types (10/16) had > 400 such upregulated PRC2^−^-CGI genes (Fig. 1b). For example, *CEP72*, the gene encoding a centrosomal protein associated with regulation of cell cycle, harbors an active promoter marked with H3K27ac and ATAC-Seq chromatin accessibility in normal colon tissue, and becomes transcriptionally upregulated in colon cancer with an increase in both H3K27ac and ATAC-Seq accessibility (Fig. 1e).

We further confirmed the chromatin state of normal tissues in the three classes of CGI genes, and Fig. 1f shows normal colon tissue as an example. As anticipated, H3K27me3 levels were similarly high in both PRC2^+^-CGI hypermethylated and PRC2^+^-CGI upregulated promoters, but undetectable in PRC2^−^-CGI promoters. This was true for other normal tissues (Supplementary Fig. 3), validating our use of ESC PRC2 markers to define the PRC2^+^-CGI classes, as they largely maintain their PRC2^+^ character during normal development. The nuclear lamina binds to a large fraction of silent heterochromatin regions, and H3K27me3 is enriched in lamina-associated domains (LADs)^25^. As anticipated, few upregulated PRC2^−^-CGI promoters in colon cancer overlapped with LADs (Fig. 1f). Interestingly, despite high H3K27me3 signal in both PRC2^+^-CGI classes, hypermethylated PRC2^+^ promoters showed much stronger enrichment in LADs than upregulated PRC2^+^ promoters (Fig. 1f). This coincided with lower expression and H3K27ac levels in the hypermethylated PRC2^+^-CGI class relative to the upregulated PRC2^+^-CGI class. Extending the LAD analysis to pan-cancer samples, we found that the hypermethylated PRC2^+^-CGI class had an average of 2.4X more genes (37.8% vs 15.7%) within LADs than the upregulated PRC2^+^-CGI class. In fact, the upregulated PRC2^+^-CGI class was more similar to the upregulated PRC^−^-CGI class, which had an average of 9.8% of genes within LADs (Fig. 1g). In a Pearson correlation analysis, the H3K27me3 level was as expected (positively) correlated with both classes of PRC2^+^-CGI promoters, but LADs were only correlated with hypermethylated PRC2^+^-CGI promoters (Supplementary Fig. 4a). Similarly, expression in normal tissue was (negatively) correlated with hypermethylated PRC2^+^-CGI promoters but not upregulated PRC2^+^-CGI promoters. While this predisposition of lowly expressed PRC2^+^-CGI genes to be hypermethylated in cancer is well-established, our results indicate that an additional feature (that is, location within a LAD) may be involved.

As described above, DNA methylation valleys (DMVs) represent a functionally important subgroup of PRC2^+^-CGI genes that have hypermethylated promoters in cancer. Looking specifically at DMVs, we found that they were associated both with hypermethylation and upregulation of PRC2^+^-CGI genes in similar ratios (Supplementary Fig. 4b, c), although hypermethylation tended to be slightly more enriched. Overall, DMVs were represented at comparable proportions in these expression classes as they were in PRC2^+^ genes overall (green bar in Supplementary Fig. 4c). Given the functional importance of these genes during development, upregulated PRC2^+^ DMV genes may represent a small but important class of cancer-promoting genes.

### Upregulated PRC2^+^-CGI genes have increased promoter H3K27ac and accessible chromatin in cancer

**Fig. 2:**
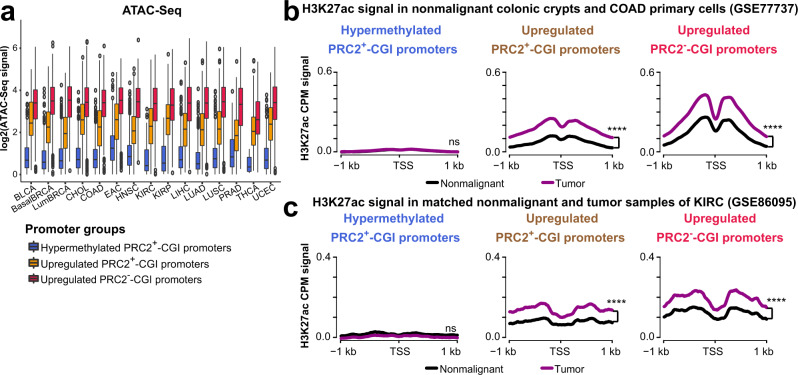
Upregulated PRC2^+^-CGI genes have increased promoter H3K27ac levels and accessible chromatin in tumors. **a** TCGA ATAC-Seq signals for each of the three CGI promoter classes in each cancer type. Box plots indicate the median (middle line), 25th, 75th percentile (box) and 5th and 95th percentile (whiskers); the promoter number (*n*) of each CGI class in each cancer type is listed in Supplementary Table 2. **b** Aggregation plots of averaged nonmalignant and primary colon cancer cells H3K27ac ChIP-Seq signals (GSE77737) for each of the three CGI promoter classes from COAD (see Supplementary Fig. 5 for details). **c** Similar line plots generated using H3K27ac ChIP-Seq signal from matched nonmalignant and tumor pairs of KIRC (GSE86095, see Supplementary Fig. 5 for details). *p*-values between two groups in panel **b**, **c** were determined by a one-sided *t*-test. *p* < 0.0001****; *p* > 0.05, ns. The exact *p*-values are shown in Supplementary Data 4.

As shown in the *LEF1* example above, upregulated PRC2^+^-CGI genes may have increases in the active signals H3K27ac and chromatin accessibility. Thus, we next systematically analyzed chromatin changes at CGI promoters in cancer, using DNA methylation data and ATAC-Seq data from TCGA, as well as H3K27ac ChIP-Seq from individual studies. The TCGA ATAC-Seq project^26^ did not include nonmalignant tissues for comparison, but in tumors we could clearly see that the hypermethylated PRC2^+^-CGI promoters were inaccessible, whereas both the upregulated PRC2^+^-CGI and PRC2^−^-CGI promoters were significantly accessible (Fig. 2a). By re-analyzing data from 4 nonmalignant colonic crypts and 18 primary colon cancer cells (GSE77737), we were able to measure the cancer-specific changes in H3K27ac for the three classes of genes (Fig. 2b and Supplementary Fig. 5a). Hypermethylated PRC2^+^-CGI promoters had undetectable levels of H3K27ac in both nonmalignant and tumor samples, whereas most upregulated PRC2^+^-CGI promoters had low H3K27ac in nonmalignant samples and a significant gain in tumors. The upregulated PRC2^−^-CGI promoters also gained H3K27ac in tumors, but were typically already higher for the mark in the nonmalignant samples. We also performed differential analysis using DiffBind^27^ and found that tumor samples had significantly stronger H3K27ac intensity than nonmalignant samples in both upregulated CGI classes (Supplementary Fig. 5a). In another cohort of seven KIRC tumors with matched adjacent nonmalignant tissues (GSE86095), similar patterns were observed across the three gene classes (Fig. 2c and Supplementary Fig. 5b).

At the DNA methylation level, as anticipated, all three classes of CGI promoters had low methylation across nonmalignant tissues (Supplementary Fig. 5c). The increased DNA methylation at PRC2^+^-CGI hypermethylated promoters was evident in tumors, while methylation levels were largely unchanged in upregulated PRC2^+^- and PRC2^−^-CGI promoters. Interestingly, methylation levels were slightly higher in upregulated PRC2^+^-CGI promoters in several cancer types. This direction of change goes counter to the usual anti-correlation between DNA methylation and expression, but is consistent with observations in another study analyzing TCGA data^18^.

### Upregulated PRC2^+^-CGI genes are characterized by high levels of cancer-type specificity and regulatory plasticity

**Fig. 3:**
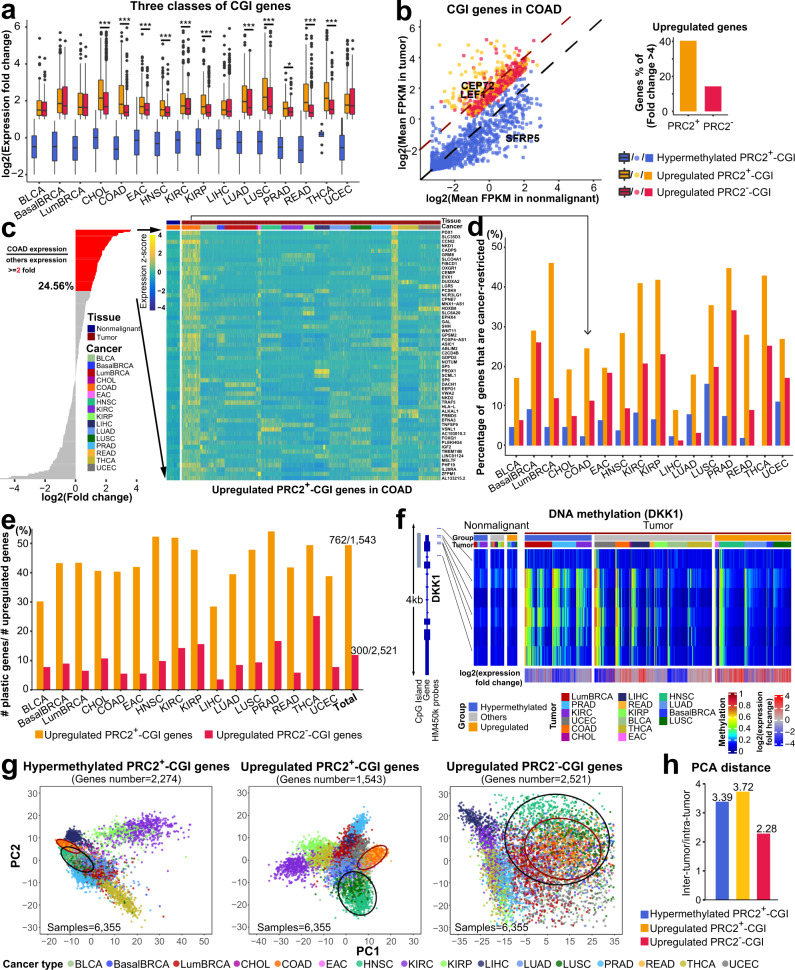
Upregulated PRC2^+^-CGI genes are characterized by high levels of cancer-type specificity and regulatory plasticity. **a** Expression fold-change between tumor and nonmalignant samples, stratified by CGI promoter classes. Expression fold-change was calculated by DESeq2. *p*-values between two upregulated groups were determined by a two-sided *t*-test. *p* < 0.001***, p < 0.01**, *p* < 0.05*. The exact *p*-values are shown in Supplementary Data 4. Box plots indicate the median (middle line), 25th, 75th percentile (box) and 5th and 95th percentile (whiskers); the gene number (*n*) of each CGI class in each cancer type is listed in Supplementary Table 2. **b** Individual genes plotted for COAD, exemplary genes from Fig. 1c–e were highlighted. **c** Cancer type-restricted genes are identified based on expression fold-change between a specific cancer type (COAD in this example) versus all other cancer types. Fold-change is shown on the left, with all genes sorted by fold-change and those with ≥2 (“cancer-type-restricted genes”) shown in red. The 56 cancer-type-restricted genes for COAD are shown as a heatmap on the right. **d** The percentage of cancer-type-restricted genes from each gene class, shown by cancer type. **e** Plastic genes were defined as those assigned to the upregulated group in one cancer type, and hypermethylated in another. The percentage of plastic genes is the number of plastic genes in each cancer type divided by the total number of upregulated genes in that cancer type. **f** TCGA methylation and expression data are shown for a plastic gene (*DKK1*). **g** PCA analyses using expression values from each of the three CGI gene classes. The black circle represents squamous cancers (LUSC, HNSC, and a subset of BLCA) and the dark red circle represents gastrointestinal cancers (EAC, COAD and READ). In order to keep the scale consistent, extreme outliers were removed: 6 samples from left, 1 from middle, and 277 from right. **h** The average PCA distance ratio of inter-tumor versus intra-tumor samples for each class of CGI genes. Intra-tumor distance: the mean distance of all tumor pairs within the same cancer type; inter-tumor distance: the mean distance of all pairs in different cancer types.

We next sought to compare expression levels for the three CGI gene classes to determine their specificity with respect to cancer type. Consistent with earlier reports^7^, hypermethylated PRC2^+^-CGI genes were slightly downregulated in TCGA tumors relative to adjacent nonmalignant tissues (Fig. 3a, b and Supplementary Fig. 6a, b). While both upregulated PRC2^+^-CGI and PRC2^−^-CGI classes were pre-selected to have an expression increase of at least 2-fold, the PRC2^+^-CGI set showed higher relative increases in 11/15 cancer types (Fig. 3a). For example in COAD, ~40% (92/228) of upregulated PRC2^+^-CGI genes were increased by more than 4-fold; in comparison, only 15% (62/424) of PRC2^−^-CGI class showed a 4-fold increase (Fig. 3b). The higher induction of PRC2^+^-CGI genes was observed in 13 cancer types (Supplementary Fig. 6c, d). This pattern held true after stratifying the upregulated genes by different normal baseline expression levels (Supplementary Fig. 6e), suggesting that the higher increase was not due to the lower baseline expression level of PRC2^+^-CGI genes in nonmalignant samples. The higher induction of a subset of PRC2^+^-CGI genes implicates their biological significance in cancer, with their expression levels potentially being under positive selection.

To investigate cancer-type specificity, we determined the extent and significance of expression differences between each cancer type vs. all others, and labeled those with fold-change ≥ 2 as cancer-type-restricted genes (Fig. 3c). While hypermethylated genes are known to have some cancer-type specificity^24^, this class had the lowest percentage of cancer-type-restricted genes in all but 2 cancer types (Fig. 3d). Upregulated PRC2^+^-CGI genes had the largest fraction of cancer-type-restricted genes across all cancer types.

Interestingly, nearly half (762/1,543) of upregulated PRC2^+^-CGI genes were assigned to the hypermethylated class in another cancer type. In contrast, only 8.5% (300/2,521) of upregulated PRC2^−^-CGI genes showed this type of regulatory plasticity, based on an analysis of hypermethylated PRC2^−^-CGI genes (see Methods). Indeed, in every cancer type, the fraction of these “plastic genes” was much higher in PRC2^+^- than PRC2^−^-CGI class (Fig. 3e). As an example, the PRC2^+^-CGI promoter of *DKK1* is hypermethylated and silenced in LumBRCA, PRAD and KIRC, but remains unmethylated and is upregulated in BasalBRCA, LUAD/LUSC, HNSC and EAC (Fig. 3f), which is consistent with earlier reports of ER-/PR-negative^28^ vs. luminal^29^ breast cancer and lung cancer^30^. Additional examples of plastic genes are shown in Supplementary Fig. 6f, g.

We next performed unsupervised clustering of all 6,355 TCGA tumor samples with PCA analysis using expression values for each of the three gene classes separately (Fig. 3g). Cancer types were separated most clearly based on the upregulated PRC2^+^-CGI class (Supplementary Fig. 7a–d), as predicted by the higher percentage of cancer-type-restricted genes in this class. While the hypermethylated PRC2^+^-CGI class also performed well in separating cancer types, the upregulated PRC2^−^-CGI class was significantly poorer, a result we quantified by calculating the ratio of distances between samples of the same cancer type (intra-tumor distances) vs. samples of different cancer types (inter-tumor distances) in Fig. 3h and Supplementary Fig. 7e. Moreover, the PCA analysis of PRC2^+^-CGI genes revealed additional patterns, which were not found by clustering based on the other two classes (Fig. 3g and Supplementary Fig. 7a–d). These included: (i) cancer subtypes derived from the same organs were correctly split into different molecular clusters, such as breast (BasalBRCA and LumBRCA), lung (LUAD and LUSC) and kidney cancer (KIRC and KIRP); ii) cancer types sharing either similar cell-of-origin or developmental lineage were correctly clustered together across different anatomical locations, such as squamous cell carcinoma (LUSC, HNSC and a subset of squamous-like BLCA) and gastrointestinal cancers (EAC, COAD and READ).

To verify that the above features of upregulated PRC2^+^-CGI genes did not reflect simply an upregulation of lowly expressed genes (which may be functionally insignificant in cancer biology), we repeated the entire above analyses by changing the cutoff of the expression of upregulated genes in tumor samples from FPKM > 1 to FPKM > 4. Importantly, PRC2^+^-CGI genes still maintained the highest levels of cancer-type specificity and regulatory plasticity (Supplementary Fig. 8).

### Upregulated PRC2^+^-CGI and PRC2^−^-CGI genes control distinct sets of biological pathways in cancer

**Fig. 4:**
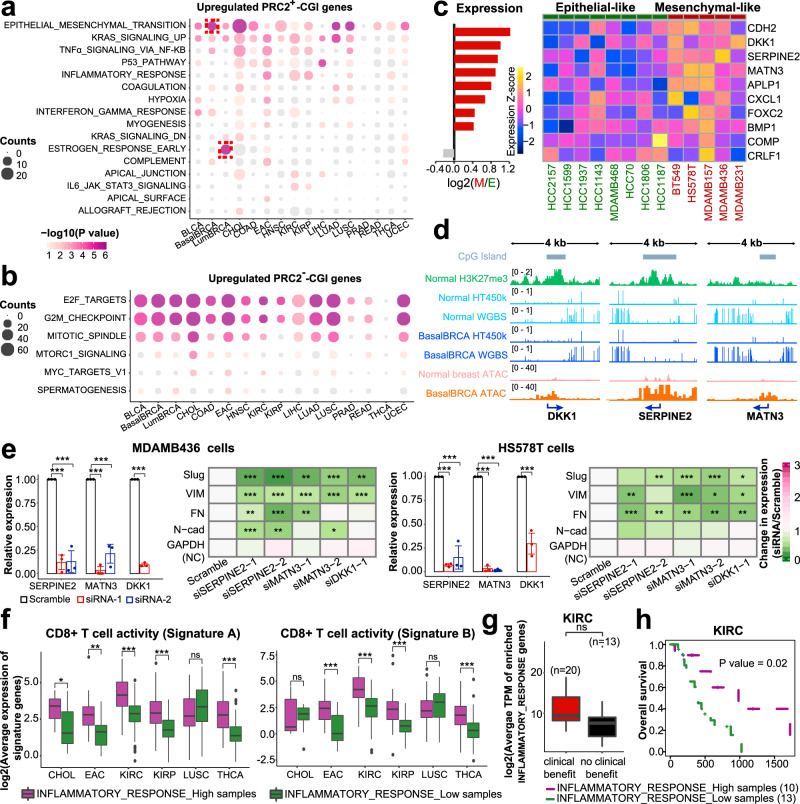
Upregulated PRC2^+^- and PRC2^−^–CGI genes control distinct sets of biological pathways in cancer. **a**, **b** Hallmark pathway enrichment results for (**a**) upregulated PRC2^+^-CGI genes and (**b**) upregulated PRC2^−^-CGI genes. “EMT” and “Estrogen response early” are highlighted in the two breast cancer subtypes. For **a**, **b** the one-sided binomial-test was performed on these two groups and the enriched hallmarks with unadjusted *p*-value < 0.05 were identified. **c** Expression of the 10 BasalBRCA upregulated PRC2^+^-CGI genes in the EMT pathway are shown in 13 BasalBRCA cell lines annotated by an established consensus EMT classification (see text). **d** IGV plots for the top 3 EMT genes from panel **c**. **e** siRNA loss-of-function assays for the top three genes followed by expression measurement of established mesenchymal markers. *n* = 3 biologically independent experiments. Data are presented as mean ± SD. *p*-values were determined by a one-sided *t*-test. *p* < 0.001***; *p* < 0.01**; *p* < 0.05*. The exact *p*-values are shown in Supplementary Data 4. **f** Average expression of CD8 + T-cell signature genes in the six cancer types enriched in the inflammatory response pathway from panel **a**, showing the top and bottom 20% of tumors based on the average expression of upregulated PRC2^+^-CGI genes in the inflammatory response pathway. Signature A contains *CCL2*, *CCL3*, *CCL4*, *CXCL9*, *CXCL10*, *CD8A*, *HLA-DOB*, *HLA-DMB*, *HLA-DOA*, *GZMK*, *ICOS* and *IRF1*^36^. Signature B contains *GZMA* and *PRF1*^37^. *p*-values were determined by a one-sided *t*-test. *p* < 0.001***; *p* < 0.01**; *p* < 0.05*; *p* > 0.05, ns. The exact *p*-values are shown in Supplementary Data 4. Expression datasets were obtained from TCGA. We only showed cancer types where the pathways were enriched with a *p*-value < 0.05. The total tumor sample sizes with expression data in different cancer types are listed in Supplementary Table 1 and the top and bottom 20% of tumor samples in each cancer type were used. **g** The average expression of upregulated PRC2^+^-CGI genes in the inflammatory response pathway in KIRC patients with differential response to immune checkpoint therapies. Expression datasets are obtained from Miao et al.^38^. *p*-value was determined by a one-sided *t*-test. Box plots in (**f**, **g**) indicate the median (middle line), 25th, 75th percentile (box) and 5th and 95th percentile (whiskers). **h** Kaplan–Meier survival plot analyzing the average expression of upregulated PRC2^+^-CGI genes in inflammatory response pathway using the same cohort of KIRC patients.

Considering that hypermethylated PRC2^+^-CGI genes have been well-studied and most have little or no expression change in cancers, here we paid more attention to the upregulated genes. We explored differential biological functions of upregulated PRC2^+^-CGI and PRC2^−^-CGI genes in each independent cancer type using Hallmark pathway enrichment, setting one of the two gene classes as the foreground and the other as the background. This revealed distinct sets of biological pathways enriched in the two classes of genes (Fig. 4a, b). Among the top-ranked PRC2^+^-CGI-enriched pathways, some were shared across multiple cancer types, including “Epithelial mesenchymal transition (EMT)”, “KRAS signaling up”, and “TNFα signaling via NF-KB” pathways, while others such as “Estrogen response early” were specific to a single cancer type (Fig. 4a). In contrast, PRC2^−^-CGI genes had little cancer-type specificity (Fig. 4b), and the three top-ranked pathways were all cell-cycle related, which were enriched across 15/16 cancer types (“E2F targets”, “G2M checkpoint” and “Mitotic spindle”, Fig. 4b). This marked difference in cancer-type specificity was even more apparent at the individual gene level, with 50–61% of PRC2^−^-CGI genes enriched in the top three pathways being shared by over half of all cancer types, compared to only 2–8% of genes enriched in the top three PRC2^+^-CGI pathways (Supplementary Fig. 9). These findings highlight that upregulated PRC2^+^-CGI genes control distinct sets of biological pathways in a cancer-type-specific manner, consistent with their high cancer-type expression specificity described above.

We next sought to functionally validate the pathway enrichment results of PRC2^+^-CGI genes, using the EMT pathway in BasalBRCA as an example. We chose this example since it was the most significantly enriched pathway across multiple cancer types, and because EMT has well-defined biological significance in BasalBRCA, which also provides multiple cell line models for experimental interrogation in vitro^31^. An established consensus classification of EMT based on expression data^32^ was used to identify epithelial- and mesenchymal-like basal breast cell lines, and we selected the 8 mesenchymal- and 5 epithelial-like cell lines that were also profiled by the Cancer Cell Line Encyclopedia (Supplementary Fig. 10a). Of the 10 PRC2^+^-CGI upregulated genes that were identified in the enriched EMT pathway in BasalBRCA, we found that 8 had higher expression in the mesenchymal- compared to the epithelial-like lines (Fig. 4c), including several known EMT-promoting factors, including *CDH2*, *CXCL1*, *FOXC2* and *BMP1*^31,33,34^. Most of these 10 genes showed clear PRC2 occupancy in normal breast tissue, as well as high chromatin accessibility in BasalBRCA TCGA tumors (Fig. 4d and Supplementary Fig. 10b).

Three of the top four genes enriched in PRC2^+^-CGI EMT pathway had not been functionally implicated in the EMT phenotype in breast cancer (*SERPINE2*, *DKK1*, *MATN3*). We performed siRNA loss-of-function assays for these genes in two mesenchymal-like cell lines (MDAMB436 and HS578T) that had high endogenous levels of these three factors (Fig. 4c). In both cell lines, knockdown of either *SERPINE2*, *DKK1,* or *MATN3* by individual siRNAs markedly reduced the expression of known mesenchymal markers (Fig. 4e) and increased the expression of epithelial markers (Supplementary Fig. 10c). These results validate the biological contribution of PRC2^+^-CGI genes to the EMT pathway and identify three PRC2^+^-CGI encoded factors (SERPINE2, DKK1, MATN3) with EMT-promoting function in basal breast cancer. As described above, *DKK1* is also notable as a plastic gene and becomes hypermethylated/silenced in LumBRCA (Fig. 3f).

In addition to the EMT pathway, we noted that two immune-related pathways were ranked among top 5 in the PRC2^+^-CGI class, namely “TNFα signaling via NF-KB” and “Inflammatory response”. Since both pathways have well-defined roles in anti-tumor immunity and contribute to immune-checkpoint blockade therapy^35^, this raises the possibility that the activation of these two pathways by PRC2^+^-CGI genes might be associated with increased immunity against cancer cells. We thus analyzed the cytotoxic activity of infiltrating CD8 + T cells based on two independent, well-established gene signatures^36,37^ in the six cancer types enriched for “Inflammatory response” (from Fig. 4a). Tumor samples with higher average expression of “Inflammatory response” PRC2^+^-CGI genes showed higher cytotoxic activity of intratumoral CD8 + T cells in most cancer types (Supplementary Fig. 10d left panel and Fig. 4f), and this was the case for most of the 16 “Inflammatory response” genes individually (Supplementary Fig. 10d right panel and Supplementary Fig. 10e). The same was true for PRC2^+^-CGI genes of the “TNFα signaling via NF-KB” pathway (Supplementary Fig. 10f, g), albeit these two pathways share a number of genes in common. We next explored whether activation of these pathways by PRC2^+^-CGI genes was associated with the response to immune-checkpoint blockade therapy. Of all the enriched cancer types, only KIRC patients had available RNA-Seq data prior to immuno-therapy^38^. Compared with patients with no clinical benefit from anti-PD-1 therapy, those showing clinical response expressed higher “Inflammatory response” PRC2^+^-CGI genes albeit without reaching statistical significance (Fig. 4g), and patients with higher expression of these genes also had better overall survival following anti-PD-1 therapy (Fig. 4h). A similar trend of TNFα pathway was also observed (Supplementary Fig. 10h, i).

### Upregulated PRC2^+^-CGI genes are linked to distal enhancers targeted by specific transcription factor binding sites (TFBSs)

**Fig. 5:**
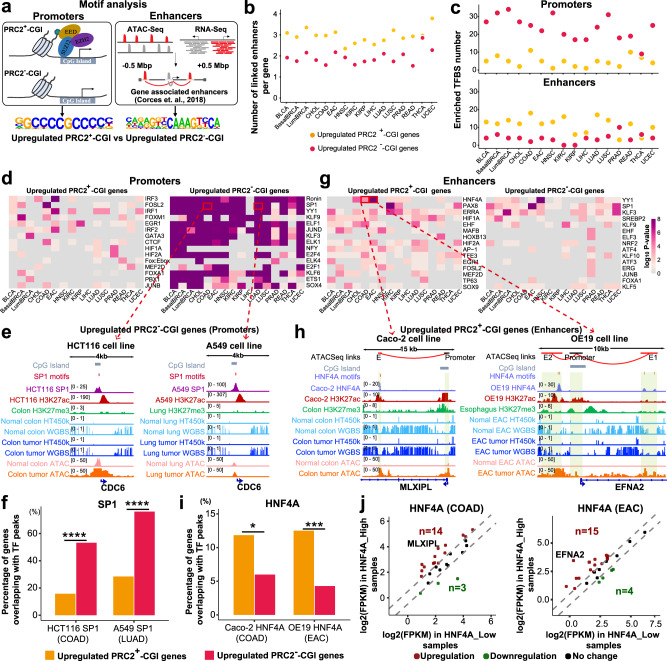
Upregulated PRC2^+^-CGI genes are linked to distal enhancers targeted by specific transcription factor binding sites (TFBSs). **a** Sequence motif enrichment analysis was performed for upregulated PRC2^+^-CGI and PRC2^−^-CGI genes using either promoter or enhancer regions. The linked enhancers are from “enhancer-to-gene links” defined by the TCGA ATAC-Seq consortium. **b** The number of linked enhancers per gene in both gene classes. **c** The number of significantly enriched TF motifs in promoter and enhancer regions. **d** The top 15 enriched TFs identified in promoter regions, selected by taking the most significant *p*-values across cancer types. **e** IGV plots showing the promoter region of *CDC6* (a PRC2^−^-CGI gene) with predicted SP1 motifs and occupied by SP1 in HCT116 COAD cancer cells (left) and A549 LUAD cells (right) by ChIP-Seq from the ENCODE project. **f** TF ChIP-Seq of SP1-binding overlapping PRC2^+^-CGI vs. PRC2^−^-CGI promoters. **g** The top 15 enriched TFs identified in enhancer regions, selected by taking the most significant *p*-values across cancer types. The one-sided hypergeometric test was performed in **c**, **d** and **g** and the enriched TFs with FPKM > 10 and unadjusted *p*-value < 0.01 in the corresponding cancer types were chosen. **h** HNF4A-binding motifs were predicted within distal enhancers for PRC2^+^-CGI genes *MLXIPL* in COAD and *EFNA2* in EAC, which were validated by HNF4A ChIP-Seq in COAD cells (Caco-2) and EAC cells (OE19). ChIP-Seq datasets were re-analyzed from GSE23436, GSE96069, E-MTAB-6858 and GSE132686. **i** TF ChIP-Seq of HNF4A-binding overlapping PRC2^+^-CGI vs. PRC2^−^-CGI enhancers, from the same COAD and EAC dataset above. **j** Expression differences between TCGA HNF4A-high and HNF4A-low EAC/COAD tumors for the HNF4A target genes having enhancers overlapped by HNF4A in EAC or COAD cells (from panel **i**). High and low tumors were those in the upper and lower quintile of HNF4A expression. The cutoff for coloring is absolute fold-change ≥ 1.5. *p*-values in **f** and **i** were determined by a two-sided F**i**sher’s exact test. *p* < 0.0001****; *p* < 0.001***; *p* < 0.01**; *p* < 0.05*. The exact *p*-values are shown in Supplementary Data 4.

We next identified candidate upstream regulators of PRC2^+^-CGI vs. PRC2^−^-CGI genes using TFBS motif enrichment analysis of promoters and enhancers. The promoter analysis used the promoter regions as described above. Unlike promoters, enhancer elements can act over a wide genomic interval and are generally not annotated. To overcome this challenge, we leveraged pan-cancer “enhancer-to-gene links” identified by the TCGA consortium based on the correlation of ATAC-Seq peaks to expression of nearby genes in the same tumors^26^ (Fig. 5a). In every cancer type, the number of enhancer elements linked to each PRC2^+^-CGI gene was larger than that linked to each PRC2^−^-CGI gene (means of 2.9 vs. 1.9, Fig. 5b). This suggested that enhancers play an important role in the regulation of PRC2^+^-CGI genes in cancer, as they do in normal development. We used HOMER to compare the frequency of TFBSs in the PRC2^+^- vs. PRC2^−^-CGI genes separately for each cancer type, by setting PRC2^+^- gene elements upregulated in that cancer type as the foreground and PRC2^−^-CGI gene elements upregulated in that cancer type as the background (or vice-versa). Considering the distinct sequence contexts between promoter and enhancer regions (most notably, the high GC content and CpG density of CGI promoters, Supplementary Fig. 11a, b), we performed separate analyses for promoters and enhancers. A notable pattern emerged from these reciprocal analyses: in promoter regions, PRC2^−^-CGI genes had enrichment for more TFBS motifs across cancer types (an average of 24 motifs for PRC2^−^-CGI and 5 motifs for PRC2^+^-CGI genes, Fig. 5c upper panel). Interestingly, the TFBSs of PRC2^−^-CGI promoters had higher CpG densities than the TFBSs of PRC2^+^-CGI promoters (Supplementary Fig. 11c), despite the overall lower CpG density of PRC2^−^-CGI promoters (Supplementary Fig. 11a). Enhancer regions showed the opposite pattern of promoter regions, with the PRC2^+^-CGI class being more enriched for enhancer motifs than the PRC2^−^-CGI class in almost all cancer types (an average of 11 motifs for PRC2^+^-CGI vs. 4 motifs for PRC2^−^-CGI, Fig. 5c bottom panel). This demonstrates a fundamentally different regulatory paradigm for the two classes of CGI promoters.

We investigated the 15 most strongly enriched TF motifs, starting with promoters. Promoter motifs were relatively non-specific across cancer types in the PRC2^−^-CGI class, while those that were enriched in the PRC2^+^-CGI class tended to be more cancer-type specific (Fig. 5d). In PRC2^+^-CGI promoters, several known cancer-type-specific TFs were observed, such as FOXM1 in BasalBRCA^39^ and GATA3 in LumBRCA^40^; nevertheless, most TFs have not been previously reported in their corresponding cancer types. In PRC2^−^-CGI promoters, a number of cell-cycle related TFs were significantly enriched across many cancer types, including SP1^41^, JUND^42^, NFY^43^, E2F4^44^, E2F1^44^ (Fig. 5d), supporting the pathway enrichment analysis, which also showed cell-cycle function among upregulated PRC2^−^-CGI genes. An example target gene *CDC6* (a cell-cycle regulator) shows promoter binding of SP1 at these motifs by ChIP-Seq in cancer cells (Fig. 5e). To validate these motif results genome-wide, we compared this ChIP-Seq data to our predictions for the SP1 motif (the highest-ranking TF with available ChIP-Seq data). As predicted, in both HCT116 (COAD) and A549 (LUAD) cells, SP1-binding events were considerably more enriched in PRC2^−^- (53.2%–76.1%) than PRC2^+^-CGI promoters (15.8%–28.4%, Fig. 5f).

We next investigated the TF motifs that were most strongly enriched in enhancers (Fig. 5g). Enhancer motifs in PRC2^+^-CGI genes tended to have the reported cancer-type-specific functions such as HNF4A in GI cancers (EAC, COAD, READ)^45^, TP63 in squamous cancers (LUSC, HNSC and a subset of BLCA)^46^, PAX8 in KIRC^47^ and UCEC^48^, etc. In contrast, PRC2^−^-CGI genes had fewer motifs overall and fewer examples corresponding to established roles in cancer. Thus, we focused on PRC2^+^-CGI enhancers, and specifically the two enriched TFs mentioned above, HNF4A and TP63, which also had publicly available ChIP-Seq data. HNF4A was most strongly enriched in GI cancers, and HNF4A enhancer-binding could be observed at upregulated PRC2^+^-CGI enhancers in GI cancer cell types (Fig. 5h). TP63 was most strongly enriched in LUSC, and an example of TP63 enhancer-binding in LUSC is shown for the *TP73* gene (Supplementary Fig. 11d). We validated that HNF4A enhancer binding specifically targeted PRC2^+^-CGI genes by calculating the number of genes with linked enhancers covered by HNF4A ChIP-Seq peaks (11.8%–12.5%), and comparing it to the number of PRC2^−^-CGI genes with such enhancers (4.3%–6.0%) (Fig. 5i). A very similar trend was observed for TP63 in LUSC (Supplementary Fig. 11e). Furthermore, the number of hypermethylated PRC2^+^-CGI genes linked to HNF4A or TP63 occupancy was even lower (0.5-2.5%), suggesting an interaction specifically between these TFs and upregulation of PRC2^+^-CGI targets (Supplementary Fig. 11e). Interestingly, these activated enhancers were only modestly enriched for the HNF4A or TP63 motif itself relative to those linked to hypermethylated PRC2^+^-CGI genes, suggesting that the binding of these TFs may be necessary but not sufficient for activation of gene targets (Supplementary Fig. 11f). We further explored the correlation between the expression of HNF4A and that of its PRC2^+^-CGI target genes (*n* = 27 in COAD, *n* = 28 in EAC; Fig. 5i) across TCGA COAD and EAC tumors. We binned tumors into HNF4A-high and HNF4A-low groups based on the top and bottom quintiles of HNF4A expression, and plotted the levels of the target genes (Fig. 5j). More than half of these PRC2^+^-CGI genes (14/27 for COAD; 15/28 for EAC) had higher expression in the HNF4A-high samples, and only 3-4 genes were lower. A similar trend of TP63 targets was observed in LUSC tumors (Supplementary Fig. 11g).

### HNF4A upregulates PRC2^+^-CGI target genes through activation of distal enhancers

**Fig. 6:**
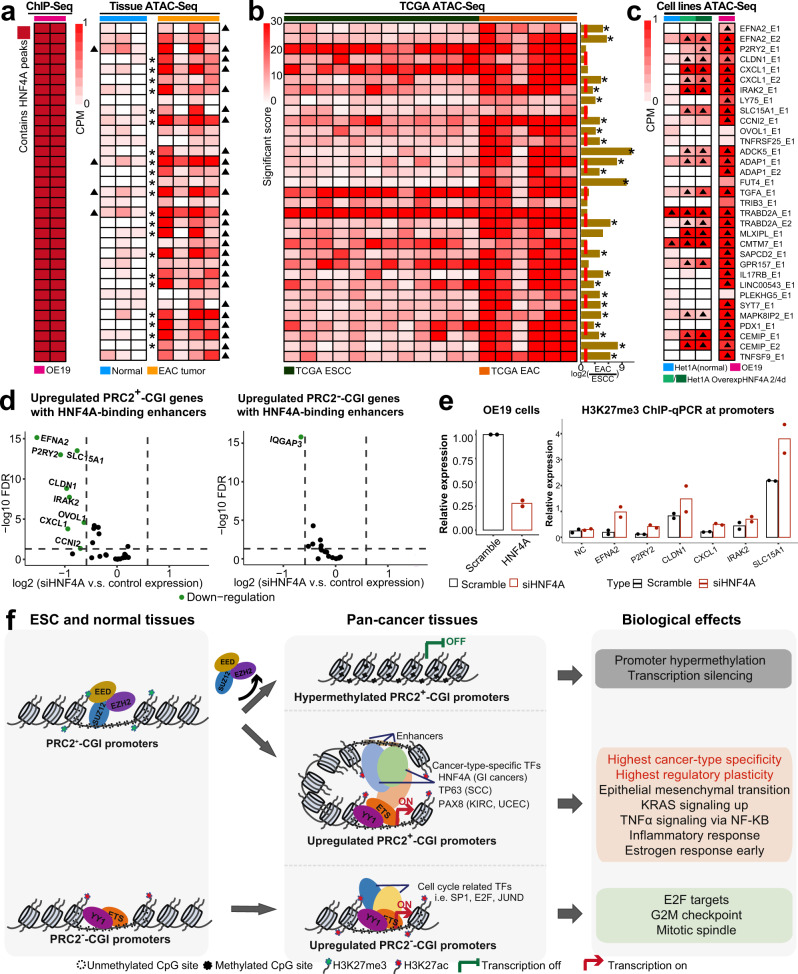
Experimental validation of HNF4A as an upstream regulator of PRC2^+^-CGI genes through activation of distal enhancers. **a**–**c** Focusing on the 33 HNF4A-binding enhancers, showing **a** HNF4A ChIP-Seq peaks (left) and ATAC-Seq peaks in EAC normal and tumor tissues (right), **b** TCGA ESCC and EAC tumor tissues, and **c** EAC cell lines. The ATAC-Seq signals in **a** are normalized with CPM method and those called as peaks are marked with a triangle. Differential analyses using DiffBind were performed to compare the difference between normal and tumors for each region in **a** and those with fold-change > 1.5 and FDR < 0.1 are marked with asterisks. TCGA ATAC-Seq signals were normalized by the TCGA consortium and the difference in ATAC-Seq signals between EAC and ESCC samples was calculated. Those genes with significant increase in EAC tumors were marked with asterisks (two-sided *t*-test, *p*-value < 0.05; The exact *p-*values are shown in Supplementary Data 4). **d** In EAC cells (OE19) with HNF4A knockdown, volcano plots show expression changes of either PRC2^+^-CGI (left) or PRC2^−^-CGI genes (right) that are linked to HNF4A-binding enhancers. **e** Promoter H3K27me3 signals were measured by ChIP-qPCR in both scramble and siHNF4A OE19 cells. *n* = 2 biological biologically independent experiments. **f** A summary graph illustrating the cancer-specific deregulation of both PRC2^+^-CGI and PRC2^−^-CGI genes, including the underlying molecular mechanisms and biological implications.

In order to better illuminate functional mechanisms, we continued to focus on the 28 PRC2^+^-CGI genes that were upregulated in EAC and had both HNF4A motifs and ChIP-Seq HNF4A-binding sites in linked enhancers. We first re-analyzed public ATAC-Seq chromatin accessibility datasets of nonmalignant esophageal epithelium and EAC tumors, focusing on these 28 PRC2^+^-CGI genes and their 33 linked enhancers. In nonmalignant esophageal epithelium, only 4 out of the 33 linked enhancers had ATAC-Seq peaks (Fig. 6a, right). In EAC tumors, 21 of the remaining 29 enhancers gained peaks (Fig. 6a, right). We performed differential analysis using DiffBind for each locus individually and found that 21/33 enhancers were significantly increased in EAC tumors vs. nonmalignant epithelium. The independent TCGA ATAC-Seq dataset (Fig. 6b) did not contain nonmalignant samples, but had both EAC and ESCC tumors, which we could utilize for comparison. In our analysis, 23 of the 33 HNF4A-occupied enhancers had significantly higher ATAC-Seq signals in EAC than in ESCC, and none had lower (Fig. 6b). We next analyzed ATAC-Seq data from normal esophageal cells following ectopic expression of HNF4A (Fig. 6c). In agreement with patient samples, 31 of the 33 HNF4A-binding enhancers were inaccessible in normal esophageal cells (Het1A), and about half (16/31) became accessible upon HNF4A overexpression.

The datasets described above demonstrate the direct regulation of these target genes by the interaction of HNF4A with the linked enhancers in an EAC-specific manner. To further validate this finding, we re-analyzed the HNF4A-wildtype and HNF4A-knockdown RNA-Seq datasets in an EAC cell line (OE19). We found that 28.5% (8/28) of the HNF4A-linked PRC2^+^-CGI genes were downregulated (Fig. 6d, left), compared to the background level for all PRC2^+^-CGI genes of 3.5% (152/4,378). Six of these 8 downregulated genes also gained ATAC-Seq peaks in the HNF4A overexpression assay in Het1A cells (Fig. 6c). Additionally, this regulation appeared to be PRC2^+^-CGI-specific—only 1 out of 17 PRC2^−^-CGI genes (5.9%) overlapping with HNF4A ChIP-Seq was downregulated in the knockdown (Fig. 6d, right).

As H3K27me3 data was unavailable for any of these cell types other than normal esophagus, we performed promoter H3K27me3 ChIP-qPCR in OE19 HNF4A-wildtype and HNF4A-knockdown cells. We performed this assay for all six genes that were downregulated upon HNF4A-knockdown and gained enhancer accessibility under HNF4A-overexpression. All gene promoters showed gain of H3K27me3 signal in the knockdown of HNF4A (Fig. 6e). Taken together, these results characterize HNF4A as an upstream regulator of PRC2^+^-CGI genes in EAC by activating distal enhancers and removing PRC2-associated H3K27me3 from the associated promoters.

## Discussion

Most PRC2-occupied promoters overlap CpG Islands and are known to be prone to de novo DNA hypermethylation and transcriptional repression in cancer, but few studies have looked systematically at expression changes in these PRC2^+^-CGI promoters and the larger class of PRC2^−^-CGI promoters^18–20^. Here we comprehensively investigated cancer-associated deregulation of all CGI promoter genes across pan-cancer samples, revealing regulatory similarities and differences between these two classes of genes. Consistent with prior findings^1,4^, we showed that many PRC2^+^-CGI genes were commonly hypermethylated and downregulated in most cancers, affecting 2,274 of 4,378 genes across in one or more of 16 cancer types. Unexpectedly, we also found a large class of PRC2^+^-CGI genes (1,543/4,378) to be upregulated in one or more cancer types. Among these upregulated PRC2^+^-CGI genes, we found many well-defined oncogenes and genes encoding tumor-promoting factors such as MYB, TWIST1, SYK, TEAD4, FOXC1, and FGFR3 (Supplementary Data 2). Previous studies in normal cells have demonstrated that PRC2^+^-CGI promoters are unmethylated^11^, with limited chromatin accessibility^49^ and weak transcriptional activity^17^. In tumors, our analysis showed that unlike hypermethylated PRC2^+^-CGI promoters, upregulated PRC2^+^-CGI promoters gain accessibility and the H3K27ac mark (illustrated in Fig. 6f). While upregulated PRC2^−^-CGI promoters also gained these active characteristics, they were much more likely to start with high baseline levels of these features in nonmalignant tissues. This helps explain why upregulated PRC2^−^-CGI genes had higher absolute expression in tumors, but PRC2^+^-CGI genes had higher fold-change differences from normal tissues (median of 3.5 for PRC2^+^-CGI vs. 2.9 for PRC2^−^-CGI genes).

Among our most intriguing findings was the high degree of cancer-type specificity in expression of upregulated PRC2^+^-CGI genes, which was higher than hypermethylated PRC2^+^-CGI and markedly higher than upregulated PRC2^−^-CGI genes. This property allowed for better clustering of cancer types and subtypes using the upregulated PRC2^+^-CGI class than either of the other two classes, although hypermethylated PRC2^+^-CGI also showed good clustering^24^. Interestingly, nearly half (762/1,543) of upregulated PRC2^+^-CGI genes were also hypermethylated in other cancer types, including some known tumor suppressors, such as *DKK1*, *NFGR*, *PRICKLE1*. For example, *DKK1* was hypermethylated in LumBRCA, PRAD and KIRC, whereas it became upregulated in BasalBRCA, LUAD/LUSC, HNSC and EAC. *DLX5* was similarly hypermethylated in LumBRCA, but it was upregulated in multiple squamous type cancers as detailed in a functional study of this gene (Manuscript under review). These findings suggest a bifurcated chromatin re-configuration of many PRC2^+^-CGI genes (“plastic” genes) during tumorigenesis, dependent on different transcriptional programs and TF activities in different cell types. This regulatory plasticity is not entirely surprising given the disproportional role PRC2^+^-CGI genes play in normal patterning and development. The regulatory complexity of PRC2^+^-CGI genes in cancer was also evident from the types of biological pathways we identified among these genes compared to other CGI genes, including important cancer-related pathways such as EMT, KRAS signalling, and TNFα signalling and inflammatory response. Indeed, these pathways were often cancer-type specific.

Functionally, upregulated PRC2^+^- and PRC2^−^-CGI genes controlled distinct sets of pathways in cancer. Specifically, upregulated PRC2^−^-CGI genes were highly enriched in cell-cycle pathways non-specifically across different cancer types. This is also consistent with the motif enrichment result that enriched TFs in the PRC2^−^-CGI class were strongly overlapped between different cancer types, and were associated with cell-cycle TFs, such as SP1, JUND, NFY, E2F4, and E2F1. Furthermore, over half of PRC2^−^-CGI genes in cell-cycle pathways were shared across cancer types, highlighting the common activation of cell-cycle-related PRC2^−^-CGI genes in cancer. In comparison, a completely different set of biological pathways were enriched in upregulated PRC2^+^-CGI genes, including EMT, KRAS signalling, and TNFα signalling and inflammatory response pathways, as mentioned above. Our finding of the involvement of a subset of PRC2^+^-CGI genes in immunologically “hot” tumors may have important implications for immune-checkpoint blockade therapy, especially given that the enzymatic activity of PRC2 can be pharmacologically targeted (e.g., by EZH2 inhibitors). Indeed, consistent with this notion, recent studies have shown that EZH2 inhibition leads to heightened anti-cancer immunity and synergizes with immune-checkpoint blockade therapy in different cancer types^50,51^.

Our conclusions above regarding cancer-type-specificity strongly suggested that the upregulated PRC2^+^-CGI genes might be controlled by distal enhancers, which govern cell-type-specific expression programs and have been shown to regulate the PRC2 status of linked promoters^52^. By using cancer type-specific enhancer links from the TCGA ATAC-Seq project^26^ combined with TFBS motif analysis, we were able to show that PRC2^+^-CGI genes were predominantly linked to specific TFBSs in distal enhancers, whereas PRC2^−^-CGI genes were linked to TFBSs in promoters (as illustrated in Fig. 6f). Unsurprisingly, the TFs whose binding sites were enriched in PRC2^−^-CGI promoters exhibited ubiquitous expression patterns across cancer types, whereas the enhancer-linked TFBSs were enriched in specific cancer types. We functionally validated a few of these TFBS/target-gene relationships using publicly available ATAC-Seq and ChIP-Seq datasets, as well as genetic perturbations. For HNF4A, a master regulator of GI cancers^45^, loss of function in cancer cells led to gain of the H3K27me3 mark at promoters and reduced expression of genes linked to HNF4A-occupied enhancers. This mode of action in cancer is consistent with the model proposed in Taberlay *et al*. for PRC2^+^-CGI gene activation during normal development^52^. While this mode of activation appears to be prevalent in cancer based on our analysis, additional layers of deregulation of these genes may be caused by genetic disruption of PRC2 proteins themselves, given the discovery of both loss-of-function and gain-of-function mutations of PRC2 complex (particularly *EZH2*) in cancer^53^.

In summary, we have systematically investigated the cancer-specific deregulation of different classes of CGI promoters, which together make up ~70% of all human promoters. We identified bifurcated deregulation of PRC2^+^-CGI genes, leading to either hypermethylation-associated gene silencing or transcriptional activation depending on the cancer type. The PRC2^+^-CGI genes that become silenced have been well-studied, but those that become activated have not, and appear to play important roles in pathways such as EMT and TNFα-associated inflammatory response in cancer. Finally, we show that many of these activating events are controlled by the activity of specific TFs in distal enhancers linked to these genes, which leads to removal of the H3K27me3 mark from linked promoters. These data together advance our mechanistic understanding of the chromatin regulation of these different gene categories in cancer, while providing a comprehensive catalog of candidate cancer-associated genes for future investigation.

## Methods

### Data sources

The TCGAbiolinks package^54^ (version 2.13.6) was used to download the sample information, mRNA expression (RNA-Seq level 3 data) and DNA methylation (Illumina HumanMethylation450 array) data of 33 types of cancers (*n* = 10,528) from the TCGA project. All the TCGA data were downloaded from GDC v16.0. Considering the distinct biology between established cancer subtypes (including esophageal adenocarcinoma vs. squamous cancer, breast luminal vs. basal cancer, lung adenocarcinoma vs. squamous cancer), they were analyzed as distinct disease subtypes. To ensure the statistical power for comparing nonmalignant and tumor tissues, cancer types with fewer than five nonmalignant samples were excluded, resulting in 16 cancer types available for analyses (Supplementary Table 1). For statistical tests, each tumor type was analyzed independently to avoid potential batch effects between TCGA disease projects. ATAC-Seq (Assay for Transposase-Accessible Chromatin using Sequencing) data of TCGA samples and pan-cancer “enhancer-to-gene” links were obtained from a recent TCGA publication^26^.

The following additional datasets were collected: H3K27ac ChIP-Seq in nonmalignant colonic crypts and primary colon cancer cells (GSE77737)^55^, H3K27ac ChIP-Seq in nonmalignant and tumor samples of kidney renal clear cell carcinoma (KIRC) from GSE86095^56^, HNF4A ChIP-Seq in OE19 (E-MTAB-6858)^57^ and Caco-2 (GSE23436) cell lines^58^, TP63 ChIP-Seq in HCC95 cell line (GSE46837)^59^, SP1 and JUND ChIP-Seq in HCT116 and A549 cell lines (ENCODE), H3K27ac ChIP-Seq in OE19 (GSE132686)^60^, HCC95 (GSE66992)^61^, HCT116 (ENCODE), Caco-2 (GSE96069)^62^ and A549 (ENCODE) cell lines. ATAC-Seq of nonmalignant colon tissue, breast epithelium and esophagus epithelium were obtained from ENCODE. We also collected ATAC-Seq datasets of nonmalignant lung tissue, lung adenocarcinoma and lung squamous cell carcinoma from NSCLC ATAC Project (https://pms.cd120.com/download.html)^63^. RNA-Seq of HNF4A knockdown, ATAC-Seq of nonmalignant esophageal epithelium, EAC tissues, normal esophageal cells (HET1A) and OE19 tumor cells were downloaded from E-MTAB-6756^57^, E-MTAB-5169^64^ and E-MTAB-6931^57^. RNA-Seq datasets from pre-treatment tumors with anti-PD-1 monotherapy in KIRC were obtained from Miao et al.^38^. We also retrieved the mRNA expression data of basal breast cancer cell lines from the Cancer Cell Line Encyclopedia (CCLE). Annotation of CGI regions was downloaded from UCSC website (http://hgdownload.soe.ucsc.edu/goldenPath/hg38/database/). LADs from normal human embryonic lung fibroblasts Tig3ET were downloaded from Guelen et al.^25^.

### Curation of CGI promoters

We extracted all transcription start sites (TSSs) from the GENCODE basic annotation file (version 31). As Illumina HumanMethylation450 array (which was used by TCGA) contains FANTOM4-annotated promoters^65^, TSSs from FANTOM4 were also integrated in our study. All the genome coordinates were converted to hg38 using the UCSC LiftOver function (https://genome.ucsc.edu/cgi-bin/hgLiftOver). We extracted the promoter regions from 250 bp upstream to 500 bp downstream (−250 to + 500 bp) of the TSSs. Promoters that are not covered by any methylation probes were excluded for further analyses. The average β-values were calculated to represent the methylation level of each promoter. We merged neighboring promoters covered by the same methylation probes and excluded those on either Y chromosome or mitochondria. Next, we used the GENCODE comprehensive annotation file (version 31) for the annotation of FANTOM4 promoters via bedtools intersect function (https://bedtools.readthedocs.io/en/latest/). Finally, only promoters overlapping with CGI regions (that is, CGI promoters) were retained for further analyses, based on the CpG Island track from the UCSC browser (Gardener-Garden criteria) (Supplementary Fig. 1).

### Identification of PRC2^+^-CGI genes in ESC (H1 cells) and normal tissues

H3K27me3 and H3K27ac ChIP-Seq profiles in both ESCs (H1) and normal tissues (colonic mucosa, lung, breast epithelium, rectum, esophagus, uterus and liver) were obtained from the combined NIH RoadMap/ENCODE data repository. Based on the 15-state epigenomic model established by Ernst et al. in ESCs^21^, we first obtained H3K27me3-positive regions by retrieving “State 10: Bivalent/Poised TSS”, “State 11: Flanking Bivalent TSS/Enhancers” and “State 13: Repressed PolyComb”. From these H3K27me3-positive regions, we identified PRC2-occupied regions by requiring them to have either EZH2- or SUZ12-binding (ChIP-Seq datasets were downloaded from ENCODE project).

As neither EZH2- nor SUZ12-binding was available in normal tissues, we next analyzed both the expression and epigenomic states of ESC PRC2^+^-CGI genes in normal tissues. An FPKM value of 4 in TCGA normal tissues readily separated PRC2^+^-CGI genes with divergent H3K27ac levels: PRC2^+^-CGI genes with FPKM < 4 had considerably lower H3K27ac signals than those with FPKM ≥ 4 (Supplementary Fig. 2a). Furthermore, we confirmed that PRC2^+^-CGI genes with FPKM < 4 had much higher H3K27me3 levels than those with FPKM ≥ 4 (Supplementary Fig. 2b). These results demonstrate that a major subset of PRC2^+^-CGI genes in ESCs (FPKM < 4) had conserved PRC2-occupancy in normal tissues, and this subset of PRC2^+^-CGI genes were selected for further analysis (Fig. 1a).

### Classification of upregulated PRC2^+^-CGI and PRC2^−^-CGI genes in cancer

Based on the raw read count matrices downloaded from TCGA, all expressed genes with (i) read counts > 0 in more than 80% of both the nonmalignant and the tumor tissues of each cancer type, and (ii) FPKM value > 1 in more than half of tumor samples, were used for differential expression analysis (Fig. 1a). DESeq2 package^66^ (version 1.22.2) was applied and those with adjusted *p*-value < 0.05 and log2 fold-change (tumor vs. nonmalignant) > 1 were considered as upregulated genes in cancer for both PRC2^+^- and PRC2^−^-CGI classes.

### Classification of hypermethylated PRC2^+^-CGI and PRC2^−^-CGI genes in cancer

To identify PRC2^+^-CGI genes with DNA hypermethylated promoters in cancer, we applied criteria based on those developed by the TCGA consortium^24^: Promoters with methylation β-values below 0.2 in >90% nonmalignant tissues and above 0.3 in over 15% tumor samples were selected (Fig. 1a). The resulting genes were additionally required to have no significant upregulation in tumor compared to nonmalignant samples, by requiring a log2 fold-change of < 1 or a *p*-value of > 0.05 (Fig. 1a). We applied the same criteria to identify the PRC2^−^-CGI genes for the PRC2^−^ plastic gene analysis.

### ChIP-Seq data analysis

Raw reads with length shorter than 51 bp were aligned to GRCh38 (ENSEMBL release 84) using Bowtie (version 1.2.2) with “--best–chunkmbs 200” option^67^. Bowtie2 (version 2.3.4.3) was applied for those reads longer than 51 bp with the “–sensitive” parameter^68^. Then the uniquely mapped reads were extracted and sorted by SAMtools (version 1.3.1) program using “-f 2 -q 10” options^69^. PCR duplicates and blacklist regions were removed by Picard MarkDuplicates tool (version 1.136, http://broadinstitute.github.io/picard/) and bedtools (version 2.27.1), respectively. ChIP-Seq peaks were called using MACS2 (Model-Based Analysis of ChIP-Seq, version 2.1.2)^70^ with the default parameters for TFs and “-q 0.01–extsize = 146–nomodel -B” options for H3K27ac. Reads were extended at default setting and normalized at -log10 of the Poisson *p-*value by MACS2 bdgcmp command using “-m ppois” option. The BedGraphToBigWig (version 4) tool was used to generate the BigWig files^71^.

### ATAC-Seq data analysis

ATAC-Seq data were analyzed using the published pipeline^26^. Bowtie2 was applied for pre-alignments to filter out reads that align to repetitive regions using “-k 1 -D 20 -R 3 -N 1 -L 20 -i S,1,0.50 -X 2000 –rg-id” parameters. For the remaining reads, Bowtie2 was used to map to GRCh38 with “–very-sensitive -X 2000–rg-id” options. Then, the SAMtools program was applied to sort and extract uniquely mapped reads, followed by the removal of PCR duplicates. Next, ATAC-Seq peaks were identified using MACS2 with “–shift -75–extsize 150 -B–nomodel–call-summits–keep-dup all -q 0.01” parameters.

### RNA-Seq analysis

For RNA-Seq datasets of control and siHNF4A in OE19 cell line, 75 bp paired-end reads were mapped to GRCh38 using HISAT2 (version 2.0.4)^72^ and counted by htseq-count program (version 0.11.2) with default parameters. Differentially expressed genes were identified by the DESeq2 package with adjusted *p*-value < 0.05 and absolute log2 fold-change (siHNF4A vs. control) > 0.5.

### Principal component analysis (PCA)

PCA was performed using the R prcomp function and point plots were generated by the ggplot2 package (version 3.1.0).

### Hallmark pathway enrichment analysis

Cancer hallmark gene sets were obtained from MSigDB^73^. For each cancer type, upregulated PRC2^+^- and PRC2^−^-CGI genes were used as the foreground and background, respectively. Then a binomial test (approximated using a *z*-test) was performed on these two groups and the enriched hallmarks with *p*-value < 0.05 were identified. To reveal the functional difference between these two groups of genes, we switched the foreground with background and repeated the same test.

### TF-binding sequence motif enrichment analysis

For the two groups of upregulated CGI promoters, sequence motif analyses were first performed using their promoter regions to identify potential TF-binding sequences through HOMER findMotifsGenome.pl script^74^ (version 4.9.1) using either one group as the foreground and the other as the background. To study enhancer regions, we used the pan-cancer “enhancer-to-gene” links from the TCGA ATAC-Seq Consortium, and performed the same motif analyses. Considering that TFs from the same TF family can recognize identical binding sequences (such as GATA and SOX families), we retained only those  motifs corresponding to TFs with FPKM > 10 and *p*-value < 0.01 in the corresponding cancer types.

### Cell culture

Breast cancer cell lines HS578T (#HTB-126) and MDAMB436 (#HTB-130) were obtained from ATCC, and EAC cells OE19 were obtained from Sigma-Aldrich, and were authenticated by the STR-PCR analysis. All cell lines used in this study were negative for mycoplasma using in-house tests. The HS578T cell line was cultured in Dulbecco’s modified Eagle medium (DMEM) (Thermo Fisher Scientific, USA) and MDAMB436 were cultured in L-15 medium with 10 µg/ml insulin (Thermo Fisher Scientific, USA) and 16 µg/ml glutathione (iCell Bioscience Inc, Shanghai). The EAC cell line OE19 was cultured in RPMI-1640 medium (Thermo Fisher Scientific, USA). All the medium was supplemented with 10% fetal bovine serum (FBS) (Omega Scientific, USA) and 100 U/ml penicillin and 100 mg/ml streptomycin (Thermo Fisher Scientific, USA).

### siRNA transfection and real-time RT-PCR

siRNA oligos were synthesized by IGEbio (IGEBio, China) and transfected into cells by Lipofectamine RNAiMAX (Thermo Fisher Scientific, USA). The cells were collected and RNA was extracted 72 h post-transfection. Real-time PCR was performed by using Power SYBR Green Master Mix (Thermo Fisher Scientific, USA). All the sequences for siRNAs and the primers for PCR were shown in Supplementary Table 3.

### Chromatin immunoprecipitation (ChIP)

OE19 cells were cross-linked with formaldehyde at final concentration of 1.42% for 15 min at room temperature and followed by 125 mM glycine for 5 min. The cells were collected and lysed with lysis buffer [150 mM NaCl, 50 mM Tris-HCl (pH 7.5), 5 mM EDTA, NP-40 (0.5% vol/vol), Triton X-100 (1.0% vol/vol)] and the nuclear pellet was collected after centrifuge by 10,000 × *g*, 1 min at 4 °C and resuspended in 1 ml shearing buffer [(20% SDS, 0.5 M EDTA (pH 8.0), 1 M Tris (pH 8.0)] for sonication. Cell debris was removed by centrifuge and the supernatant was diluted with 5-fold volume dilution buffer [(20% SDS, 0.5 M EDTA (pH 8.0), 1 M Tris (pH 8.0), 1% Triton X-100, 5 M NaCl]. 2% of the lysate was used as input and the rest was incubated with 5 μg rabbit anti-H3K27Me3 antibody (#ab6002, Abcam Biotechnology, UK) at 4 °C overnight. The next day, protein G-coupled magnetic beads (Thermo Fisher Scientific, USA) were added to pulldown protein-DNA complex. After washing the precipitated complex with lysis buffer, the complex was eluted with buffer (150 μl 0.5 M NaHCO3, 50 μl 20% SDS) and subsequently subject to de-crosslink with supplement of 8 μl 5 M NaCl at 65 °C overnight. DNA was extracted after the complex was treated with RNase A and proteinase K (Thermo Fisher Scientific, USA) and used for real-time PCR. The primers for PCR are provided in Supplementary Table 3.

### Reporting summary

Further information on research design is available in the Nature Research Reporting Summary linked to this article.

## Supplementary information

Supplementary Information

Description of Additional Supplementary Files

Supplementary Data 1

Supplementary Data 2

Supplementary Data 3

Supplementary Data 4

Reporting Summary

## Data Availability

The ChIP-Seq data used in this study are available in GEO database under accession code GSE77737, GSE86095, GSE23436, GSE46837, GSE132686, GSE66992 and GSE96069 ArrayExpress database under accession code E-MTAB-6858^57^ and ENCODE project [https://www.encodeproject.org/]. The ATAC-Seq data used in this study are available in ArrayExpress database under accession code E-MTAB-5169 and E-MTAB-6931; ENCODE project [https://www.encodeproject.org/] and NSCLC ATAC Project [https://pms.cd120.com/index.html]. The RNA-Seq data in this study are available in ArrayExpress database under accession code E-MTAB-6756. The sample information, mRNA expression (RNA-Seq level 3 data), and DNA methylation (Illumina HumanMethylation450 array) data of 33 types of cancers (*n* = 10,528) are available in TCGA project (GDC v16.0) [https://portal.gdc.cancer.gov/]. The mRNA expression data of basal breast cancer cell lines is available in CCLE [https://portals.broadinstitute.org/ccle]. Annotation of CGI regions is available in UCSC website [http://hgdownload.soe.ucsc.edu/goldenPath/hg38/database/]. The remaining data are available within the Article, Supplementary Information, or available from the authors upon request.

## Code availability

No unpublished code was used in this manuscript.
