# Publisher Correction: A pan-cancer analysis of CpG Island gene regulation reveals extensive plasticity within Polycomb target genes

**DOI:** 10.1038/s41467-021-24389-x

**Published:** 2021-06-28

**Authors:** Yueyuan Zheng, Guowei Huang, Tiago C. Silva, Qian Yang, Yan-Yi Jiang, H. Phillip Koeffler, De-Chen Lin, Benjamin P. Berman

**Affiliations:** 1 Department of Medicine, Samuel Oschin Comprehensive Cancer Institute, Cedars-Sinai Medical Center, Los Angeles, CA USA; 2 Department of Pathology, Shantou University Medical College, Shantou, Guangdong People’s Republic of China; 3 Center for Bioinformatics and Functional Genomics, Cedars-Sinai Medical Center, Los Angeles, CA USA; 4 Department of Developmental Biology and Cancer Research, Institute for Medical Research Israel-Canada, Hebrew University-Hadassah Medical School, Jerusalem, Israel

**Keywords:** Cancer genomics, Epigenomics

Correction to: *Nature Communications* 10.1038/s41467-021-22720-0, published online 30 April 2021.

In this Article there was an error in the Author contribution statement. The final sentence of the statement ‘The last two authors (D.-C.L. and B.P.B.) are co-senior authors who jointly supervised the work, and they have the right to list their names last in their CV.’ was omitted.

This has been corrected in the pdf and HTML versions of the Article.

