# Supplementary material for: A pan-cancer analysis of CpG Island gene regulation reveals extensive plasticity within Polycomb target genes
Source: Nat Commun. 2021 Apr 30;12:2485. doi: 10.1038/s41467-021-22720-0 (PMC8087678; doi:10.1038/s41467-021-22720-0)
Supplement: Supplementary file 1 — Supplementary Information [file 41467_2021_22720_MOESM1_ESM.pdf]

Supplementary Information

A pan-cancer analysis of CpG Island gene regulation reveals extensive plasticity within Polycomb target genes

Yueyuan Zheng[1,†], Guowei Huang[1,2,†], Tiago C. Silva[3], Qian Yang[1], Yan-Yi Jiang[1], H Phillip Koeffler[1], De-Chen Lin[1,*], Benjamin P. Berman[4,*]

[1]Department of Medicine, Samuel Oschin Comprehensive Cancer Institute, Cedars-Sinai Medical Center, Los Angeles, CA, USA

[2]Department of Pathology, Shantou University Medical College, Shantou, Guangdong, 515041, P.R. China

[3]Center for Bioinformatics and Functional Genomics, Cedars-Sinai Medical Center, Los Angeles, CA, USA

[4]Department of Developmental Biology and Cancer Research, Institute for Medical Research Israel-Canada, Hebrew University-Hadassah Medical School, Jerusalem, Israel

Supplementary figures and legends

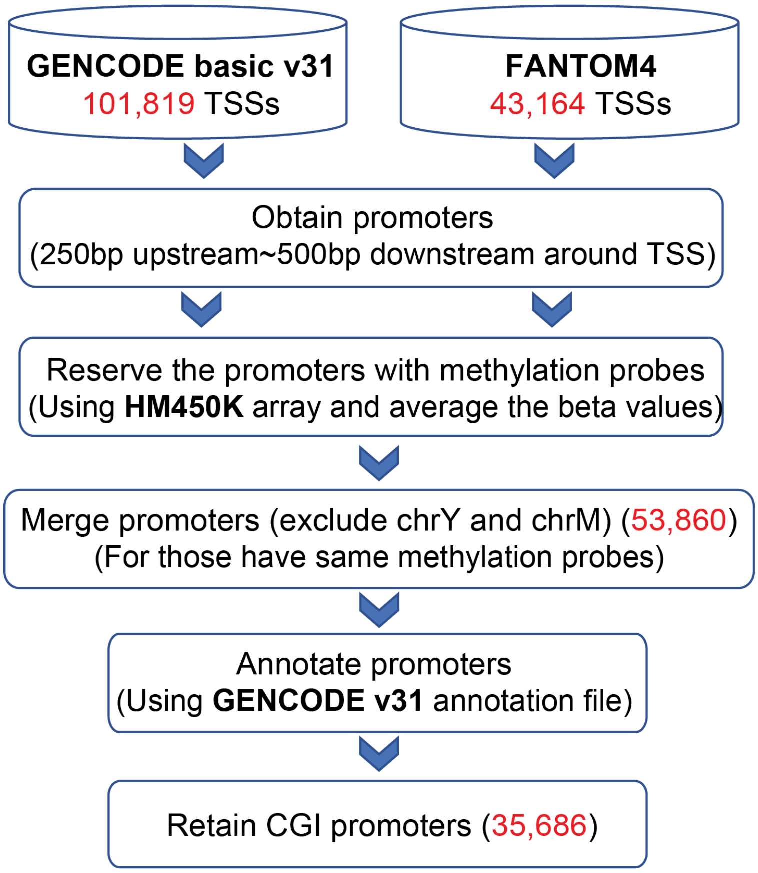

Supplementary Figure 1. The workflow of obtaining the CGI promoters. Comprehensive TSS sets were obtained from the GENCODE basic annotation file and FANTOM4. The promoter regions were extracted from 250bp upstream to 500bp downstream of the TSSs. Promoters which are not covered by any methylation probes were excluded and the average β values were used to represent the methylation level of each promoter. We then merged neighboring promoters covered by the same methylation probes and excluded those on either Y chromosome or mitochondria. The GENCODE comprehensive annotation file was used for the annotation of FANTOM4 promoters and only CGI promoters were retained for further analyses.

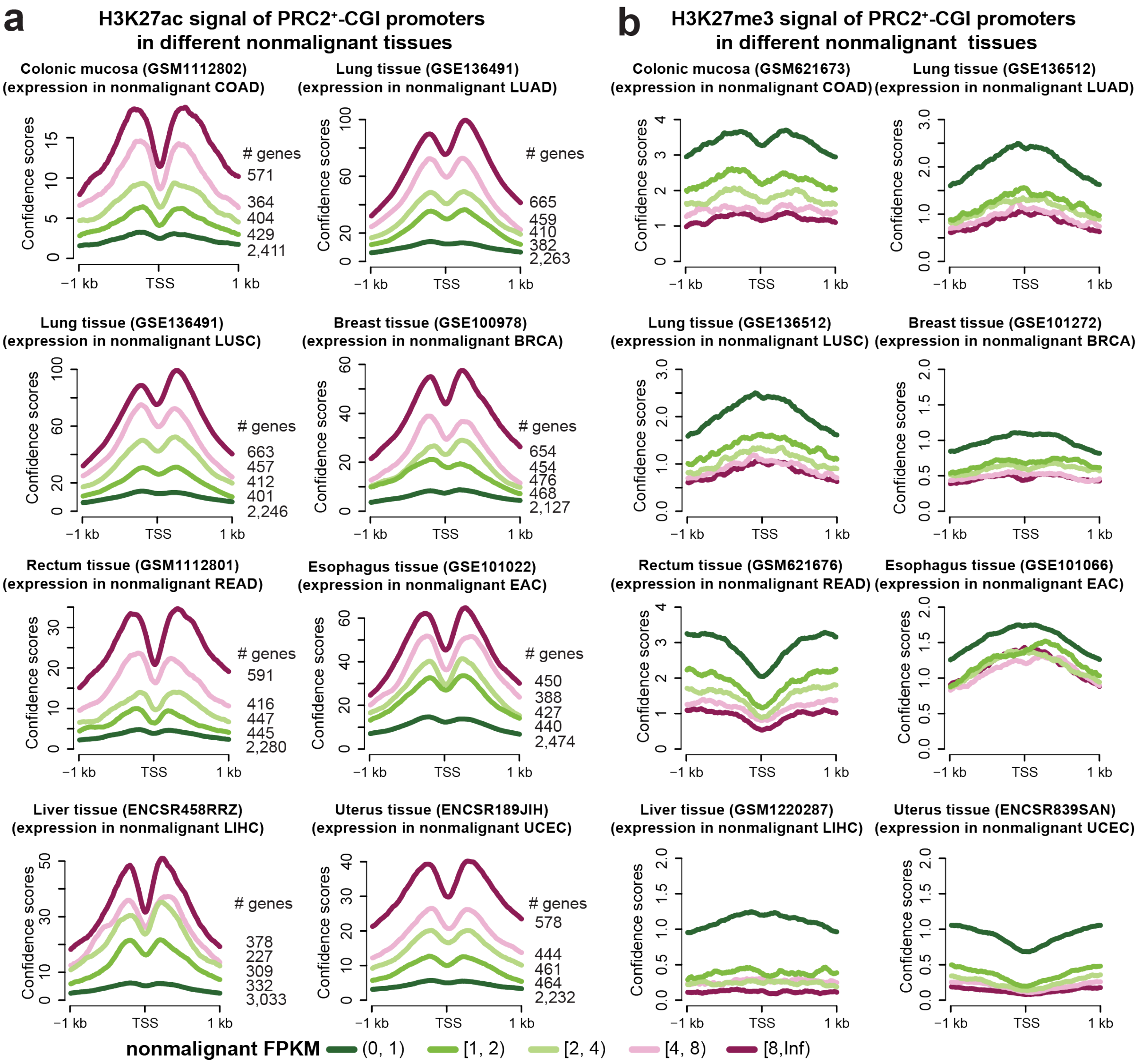

Supplementary Figure 2. Histone chromatin profiles of PRC2⁺-CGI promoters in different normal tissues. (a) H3K27ac and **(b)** H3K27me3 profiles of PRC2⁺-CGI promoters. PRC2⁺-CGI promoters are stratified into 5 groups based on gene expression in TCGA normal tissues of the corresponding cancer type. The numbers of genes in each subgroup are listed on the right side of each line in panel **a**.

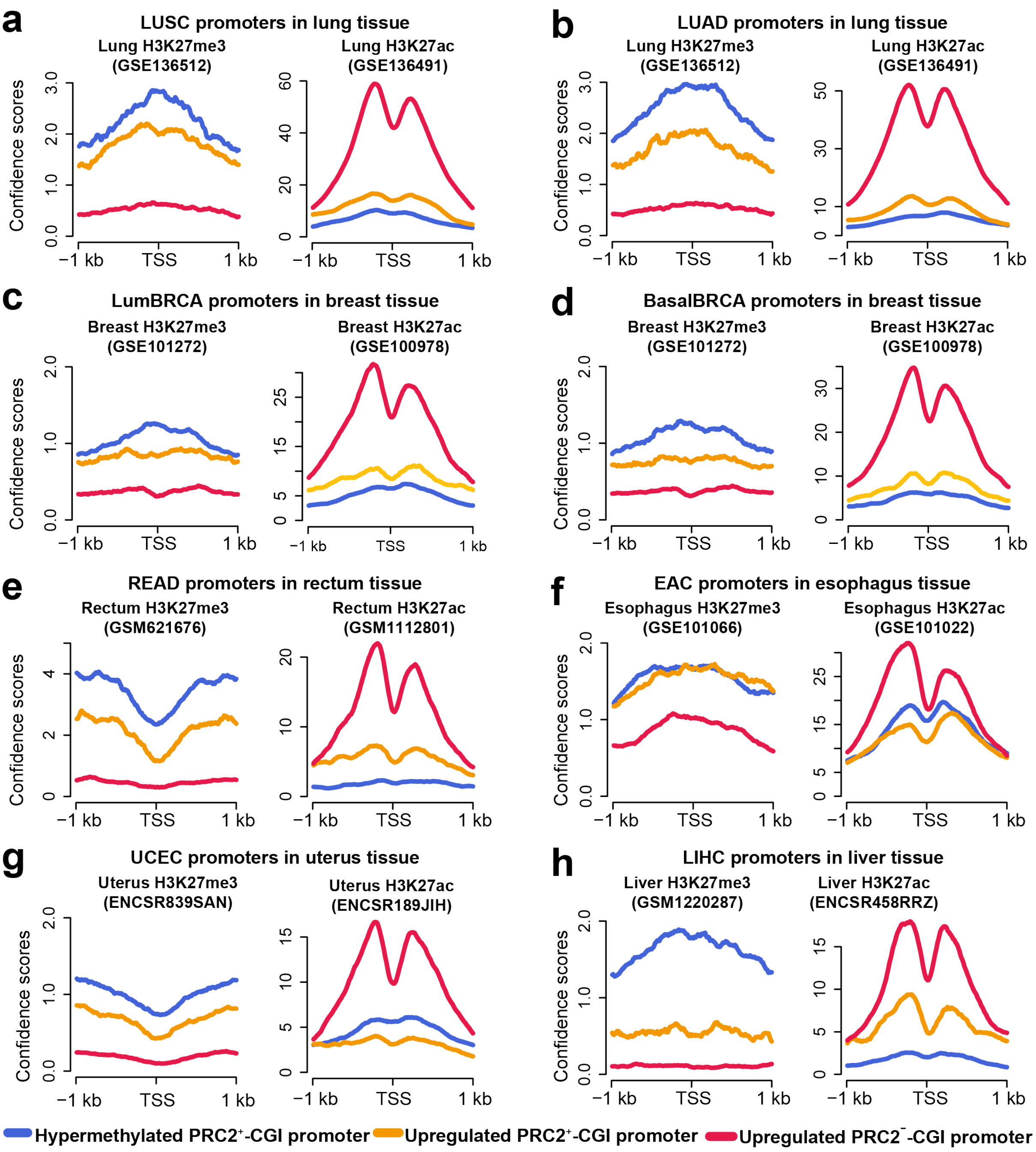

Supplementary Figure 3. Different normal tissues harbor similar H3K27me3 and H3K27ac signal patterns on the three classes of CGI promoters. (a-h) The H3K27me3 and H3K27ac signal patterns of three classes of genes identified in LUSC **(a)**, LUAD **(b)**, LumBRCA **(c)**, BasalBRCA **(d)**, READ **(e)**, EAC **(f)**, UCEC **(g)** and LIHC **(h)** were shown in the responding normal tissues.

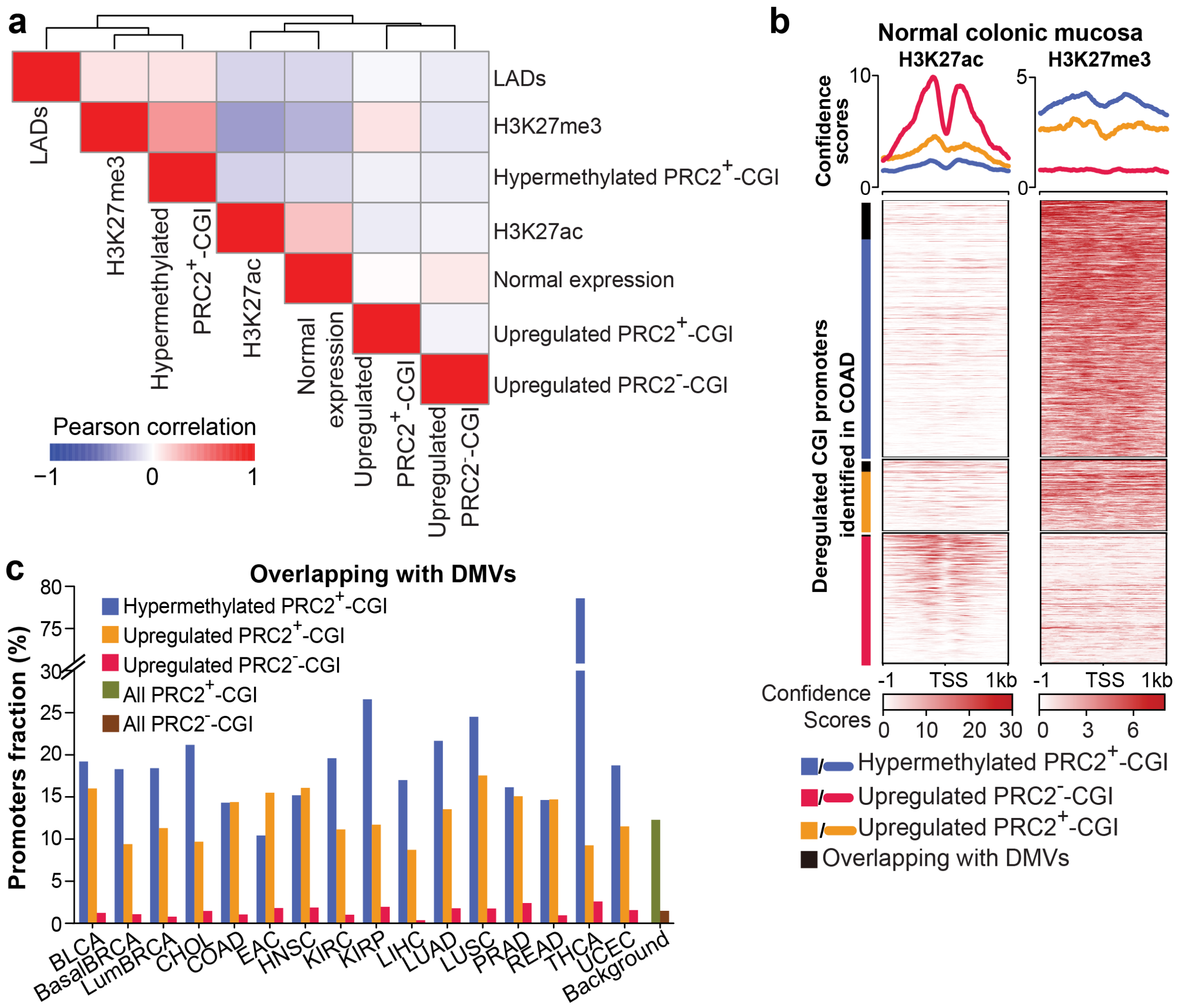

Supplementary Figure 4. Systematic identification of transcriptionally deregulated PRC2⁺-CGI and PRC2⁻-CGI genes across human cancers. (a) Pearson correlation heatmap showing the correlation among H3K27me3 signal, H3K27ac signal, gene expression in nonmalignant colon samples (normal expression), different CGI groups and LADs. The correlation matrix was calculated using all CGI promoters with FPKM<4 in nonmalignant colon samples. **(b)** Three classes of genes identified in COAD show different H3K27ac and H3K27me3 patterns in normal colonic mucosa. Promoters overlapping with ESC DNA Methylation Valleys (DMVs) from Xie et al.[1] are marked with black bars. **(c)** Fraction of all promoters overlapping DMV regions.

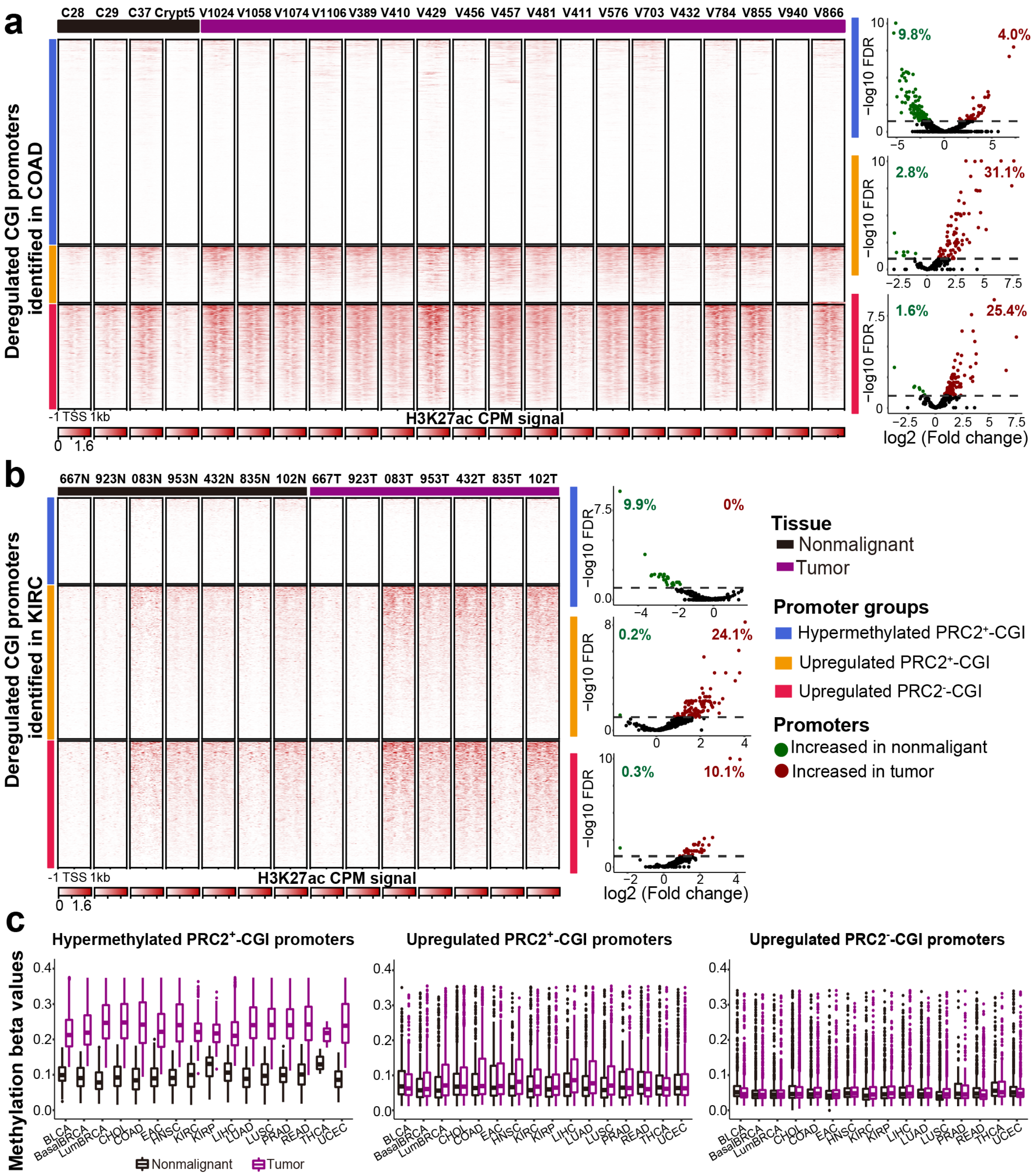

Supplementary Figure 5. Upregulated PRC2$^+$-CGI genes have increased promoter H3K27ac levels and slight methylation changes in tumors. (a-b) H3K27ac profiles of three different groups of CGI promoters in tumor and nonmalignant tissues from **(a)** COAD (GSE77737) and **(b)** KIRC (GSE86095) samples. Differential analyses using DiffBind were performed on each consensus promoter region for each CGI class and shown as volcano plots. The significant cutoff is absolute fold change>1.5 and FDR<0.1. 7 tumor samples were removed as the number of reads is smaller than 10M in panel **a**. 3 pairs of nonmaligant and tumor samples were removed due to insufficient number of peaks in panel **b**. **(c)** Methylation beta values for each of the three CGI promoter classes in nonmalignant and tumor samples

across TCGA cancer types. The top 5% of outliers in each group were outside this range and not plotted. Box plots indicate median (middle line), 25th, 75th percentile (box) and 5th and 95th percentile (whiskers); the promoter number (n) of each CGI class in each cancer type were listed in **Supplementary Table 2**.

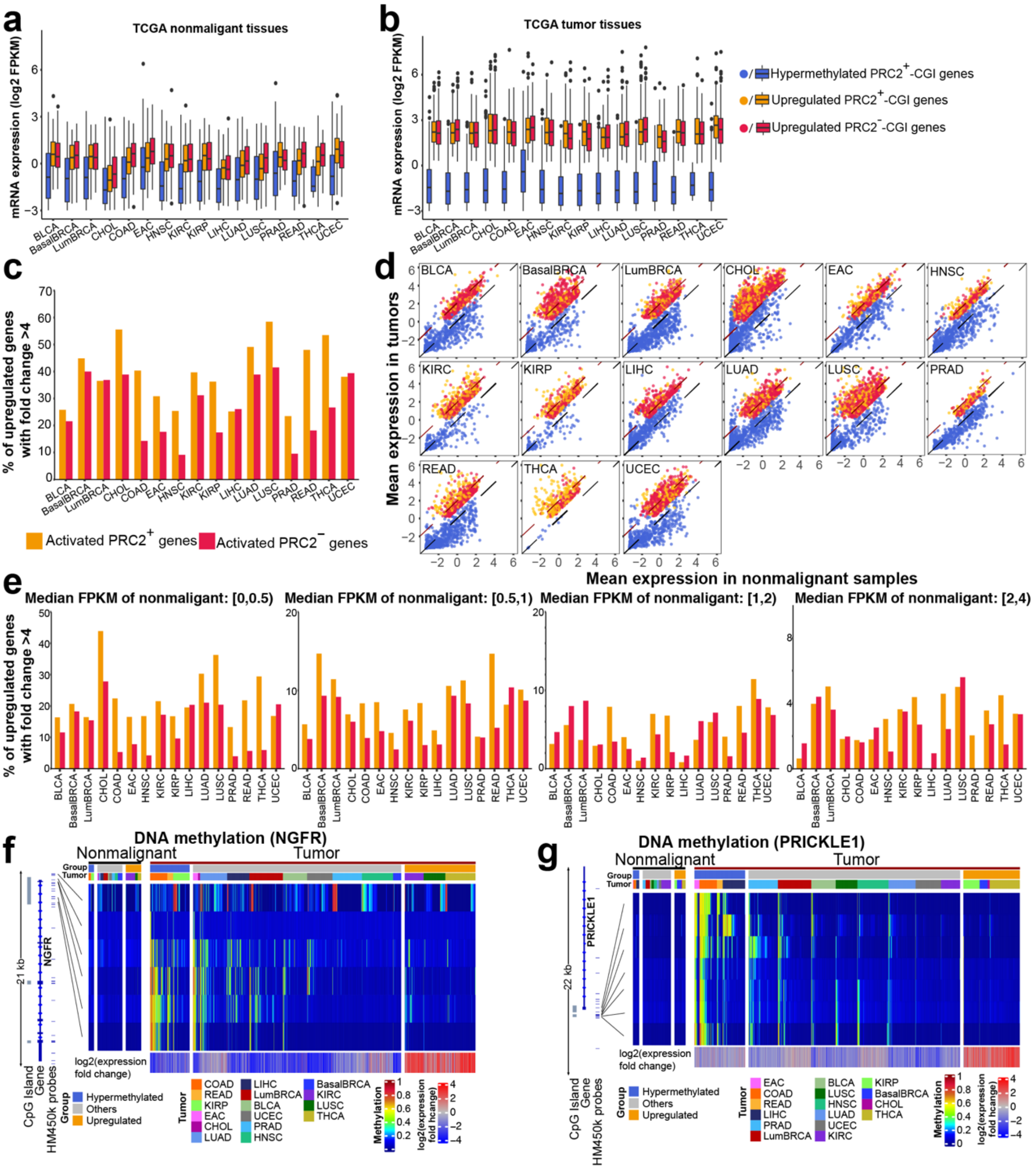

Supplementary Figure 6. Upregulated PRC2$^+$-CGI genes have the highest cancer-type-specificity and regulatory plasticity. (a-b) Pan-cancer gene expression profiles of **(a)** nonmalignant and **(b)** tumor tissues across three CGI gene categories. Box plots show the

median (middle line), 25th, 75th percentile (box) and 5th and 95th percentile (whiskers); the gene number (n) of each CGI class in each cancer type are listed in **Supplementary Table 2**. **(c)**The percentage of upregulated genes that are induced more than 4 folds compared with nonmalignant samples for all upregulated genes. Expression fold change is calculated by DESeq2. **(d)** Individual genes from three CGI classes plotted for each cancer type. **(e)** Similar with panel **c,** the upregulated genes are stratified by different expression baselines in nonmalignant samples**. (f-g)** *NFGR* **(f)** and *PRICKLE1* **(g)** are additional examples of plastic PRC2^{+}-CGI genes.

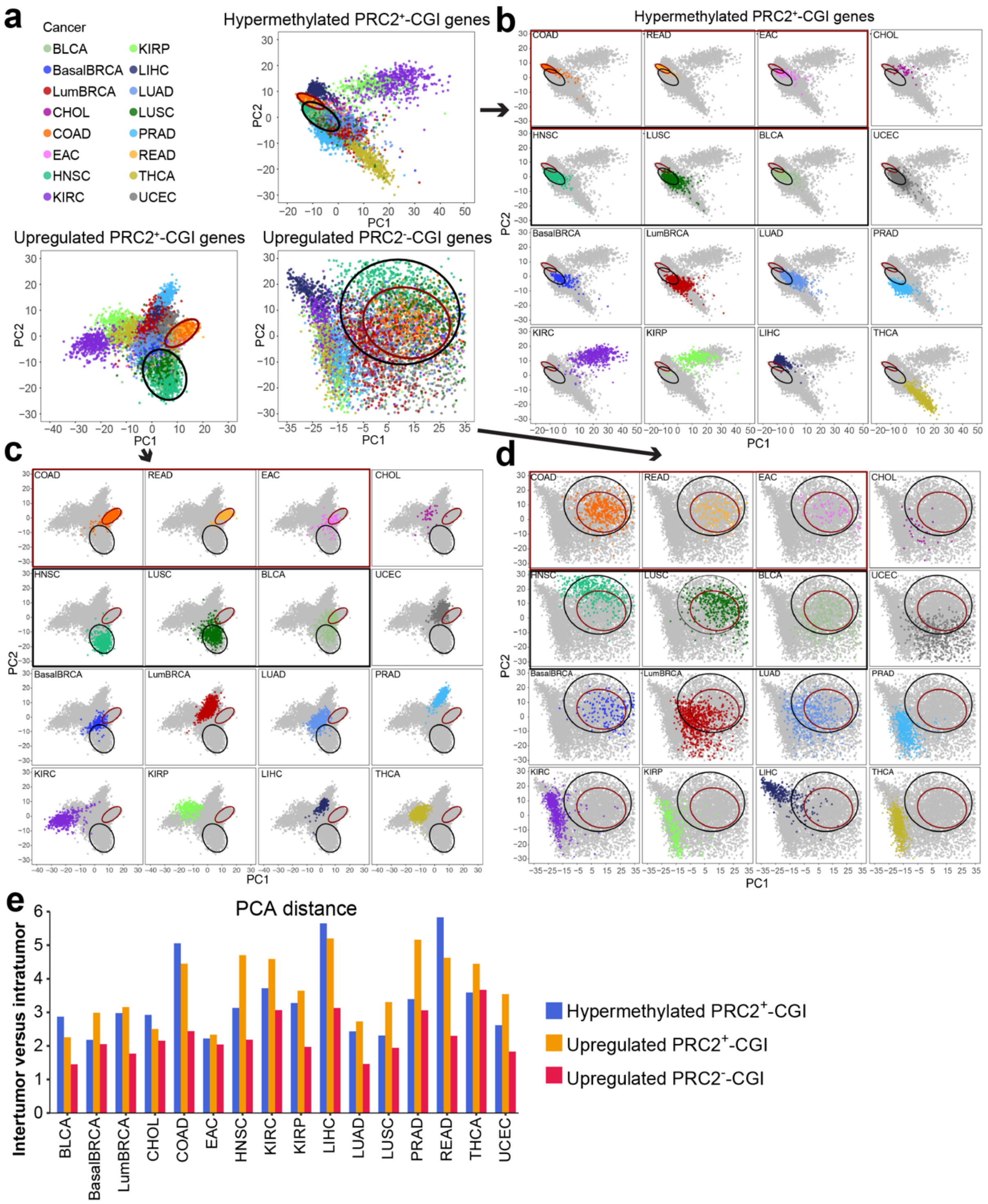

Supplementary Figure 7. Unsupervised clustering of all TCGA tumor samples with PCA analysis. (a) PCA analyses using the mRNA expression values from each of the three classes of CGI genes. The black circle denotes squamous cancers (LUSC, HNSC and a subset of BLCA) and the dark red circle denotes GI cancers (EAC, COAD and READ). **(b-d)** Each cancer type is plotted individually for each class of CGI genes. **(e)** The PCA distance ratio of inter-tumor versus intra-tumor samples across cancer types for each class of CGI genes.

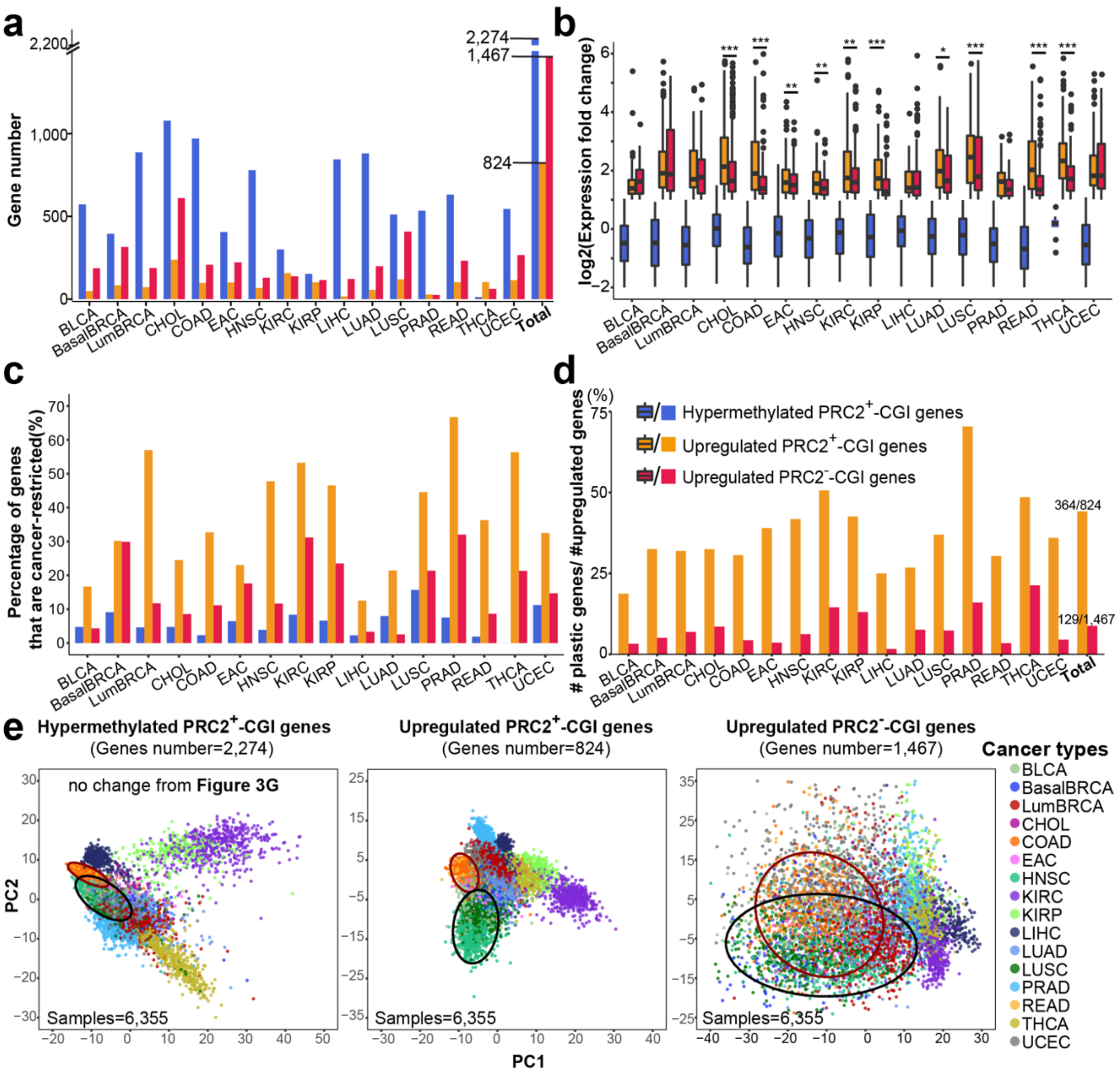

Supplementary Figure 8. Re-analyzing upregulated CGI genes selected by different expression cutoffs (FPKM>1 to FPKM>4). This criterion only changes the number of two upregulated CGI classes, but not the hypermethylated PRC2$^+$-CGI genes. For a better and integrated comparison of these three groups, the results of hypermethylated PRC2$^+$-CGI genes, which are the same as **Figure 3**, are also incorporated. **(a)** The numbers of each class of CGI genes in TCGA cancer types. **(b)** Expression fold changes between tumor and nonmalignant samples, stratified by CGI promoter classes. P values between two upregulated groups were determined by a two-sided t-test. p<0.001, ***; p<0.01, **; p<0.05, *. The exact P values are shown in **Supplementary Data 4.** Box plots show the median (middle line), 25th, 75th percentile (box) and 5th and 95th percentile (whiskers); the gene number (n) of each CGI class in each cancer type were listed in panel **a**. **(c)** The percentage of cancer-type-restricted genes from each gene class. **(d)** The percentage of plastic genes is stratified by cancer type where upregulation occurs. **(e)** PCA analyses using expression values from each of the three

CGI gene classes. Cancer types were separated most clearly based on the upregulated PRC2^{+}-CGI class. In order for better presentation while keeping the scale consistent, extreme outliers were removed: 6 samples from left, 47 from right.

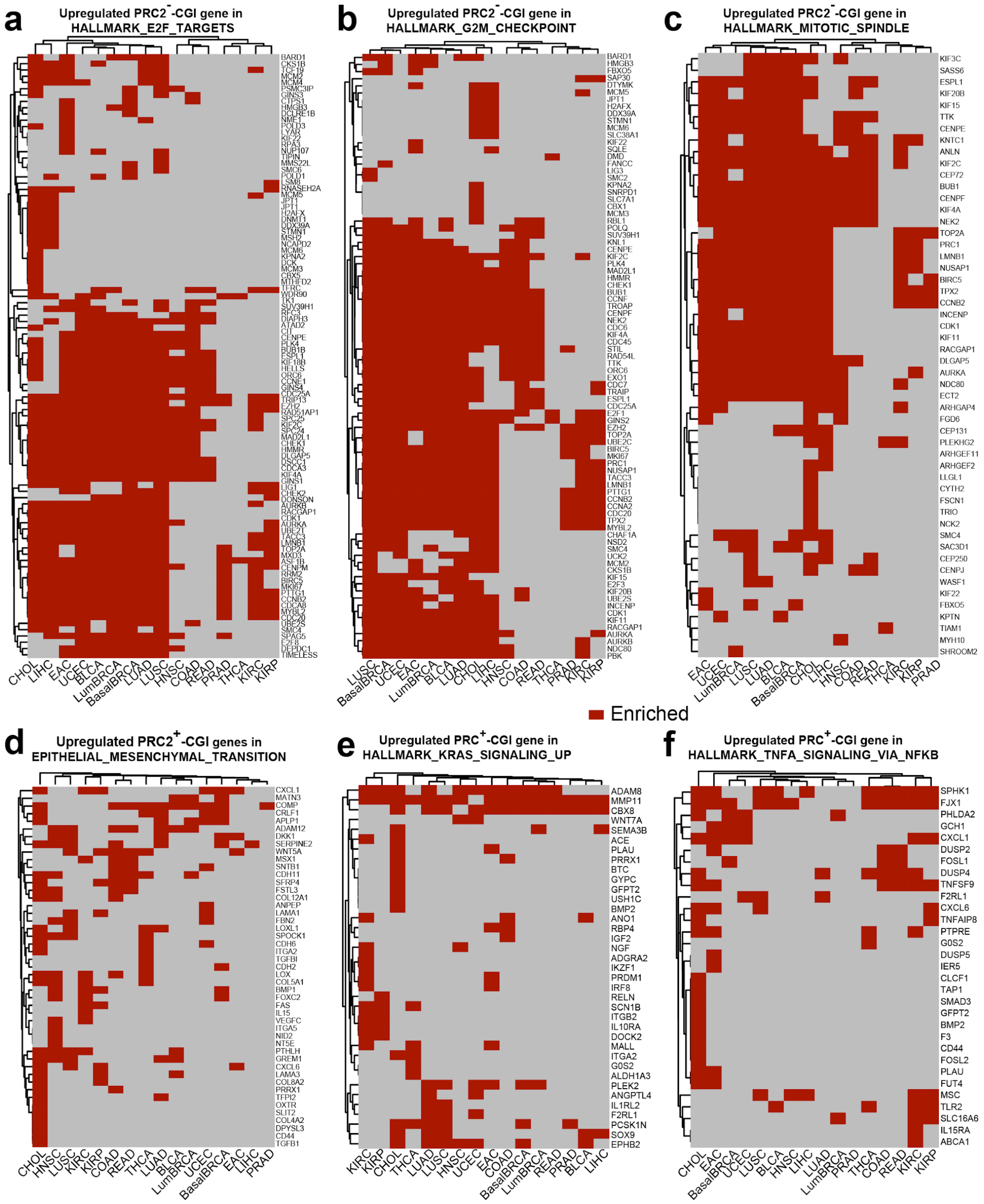

Supplementary Figure 9. Heatmaps of the upregulated PRC2^{-}-CGI genes in E2F targets, G2M checkpoint as well as mitotic spindle pathways (a-c) and PRC2^{+}-CGI genes in

Epithelial mesenchymal transition, KRAS signaling up and TNFα signaling via NFKB pathways (d-f) across cancer types. Red bar indicates the enrichment of the gene in the pathway.

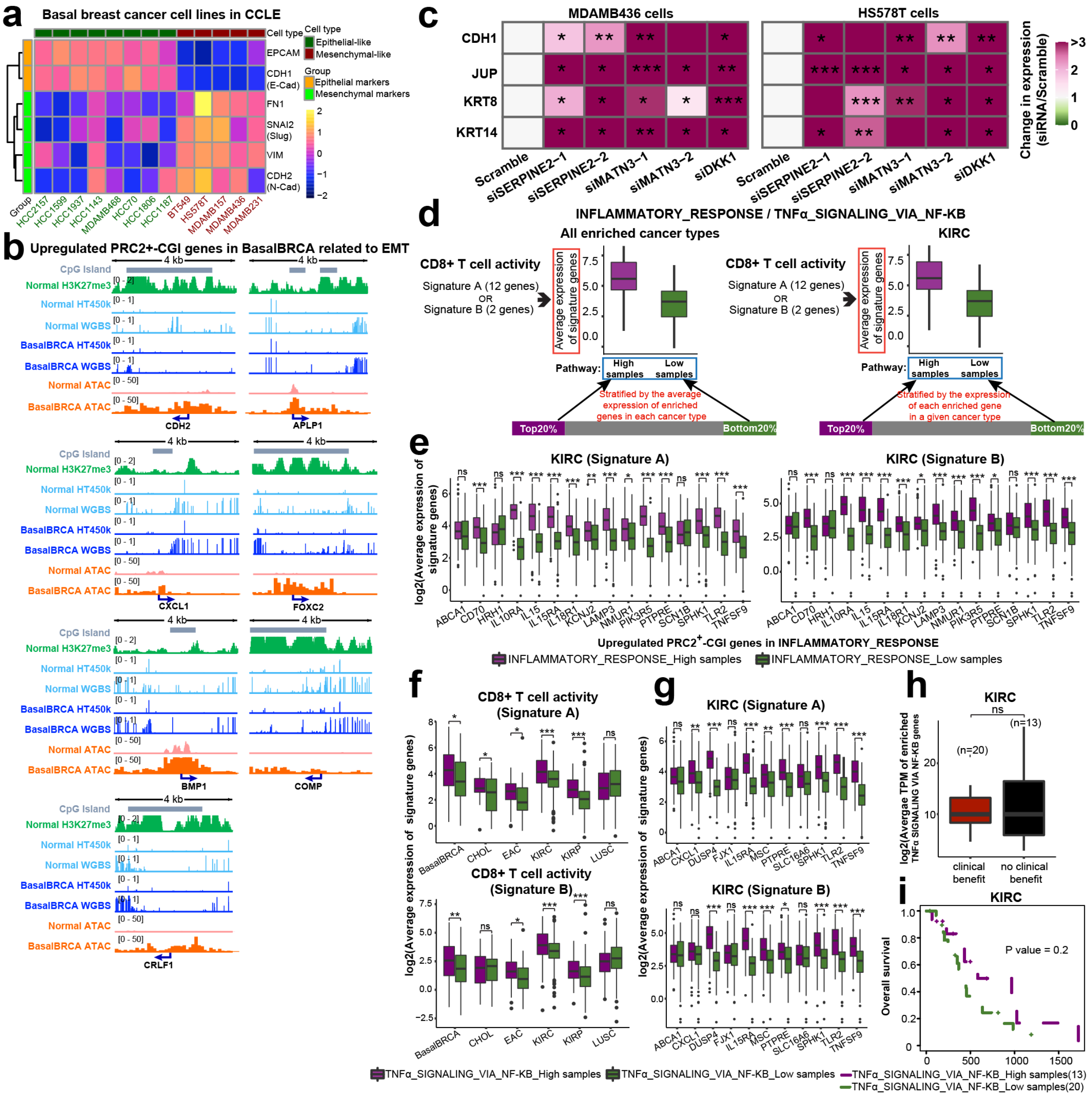

Supplementary Figure 10. Upregulated PRC2$^+$- and PRC2$^-$-CGI genes control distinct sets of biological pathways in cancer. (a) Known EMT markers were used to confirm the EMT state of epithelial-like and mesenchymal-like cells. **(b)** IGV plots show the remaining upregulated EMT-related genes in BasalBRCA. **(c)** siRNA loss-of-function assays for candidate PRC2$^+$-CGI genes followed by expression measurement of established epithelial markers. n= 3 biologically independent experiments. Heatmap shows fold change with P values for each individual gene and replicate, as determined by a one-sided t-test. p<0.001,

***; $p<0.01$, **; $p<0.05$, *. The exact P values are shown in **Supplementary Data 4. (d)** Diagrams showing the strategies to analyze the correlation between CD8+ T cell activity and immune related pathways (“Inflammatory response pathway” and “TNFα signaling via NF-KB pathway”). **(e)** In KIRC, box plots showing average expression of CD8+ T-cell signature genes in KIRC samples stratified by the expression of each enriched gene in the inflammatory response pathway. **(f-g)** Similar analyses were performed on six cancer types enriched in “TNFα signaling via NF-KB” pathway, showing the top and bottom 20% of tumors based on either the average **(f)** or the individual gene-level **(g)** expression of upregulated $PRC2^{+}$-CGI genes. For all plots **e-g**, P values were determined by a one-sided t-test. $p<0.001$, ***; $p<0.01$, **; $p<0.05$, *; $p>0.05$, ns. The exact P values are shown in **Supplementary Data 4.** For all plots **e-g**, the total tumor sample numbers (n) with expression data in different cancer types are listed in **Supplementary Table 1** and the top and bottom 20% of tumor samples were used to stratify the two groups. **(h)** The average expression of upregulated $PRC2^{+}$-CGI genes in the “TNFα signaling via NF-KB” pathway in KIRC patients with differential response to immune checkpoint therapies. Expression datasets are obtained from Miao *et.al.*[2] P value was determined by a one-sided t-test. Box plots show the median (middle line), 25th, 75th percentile (box) and 5th and 95th percentile (whiskers). **(i)** Kaplan-Meier survival plot analyzing the average expression of upregulated $PRC2^{+}$-CGI genes in “TNFα signaling via NF-KB” pathway using the same cohort of KIRC patients.

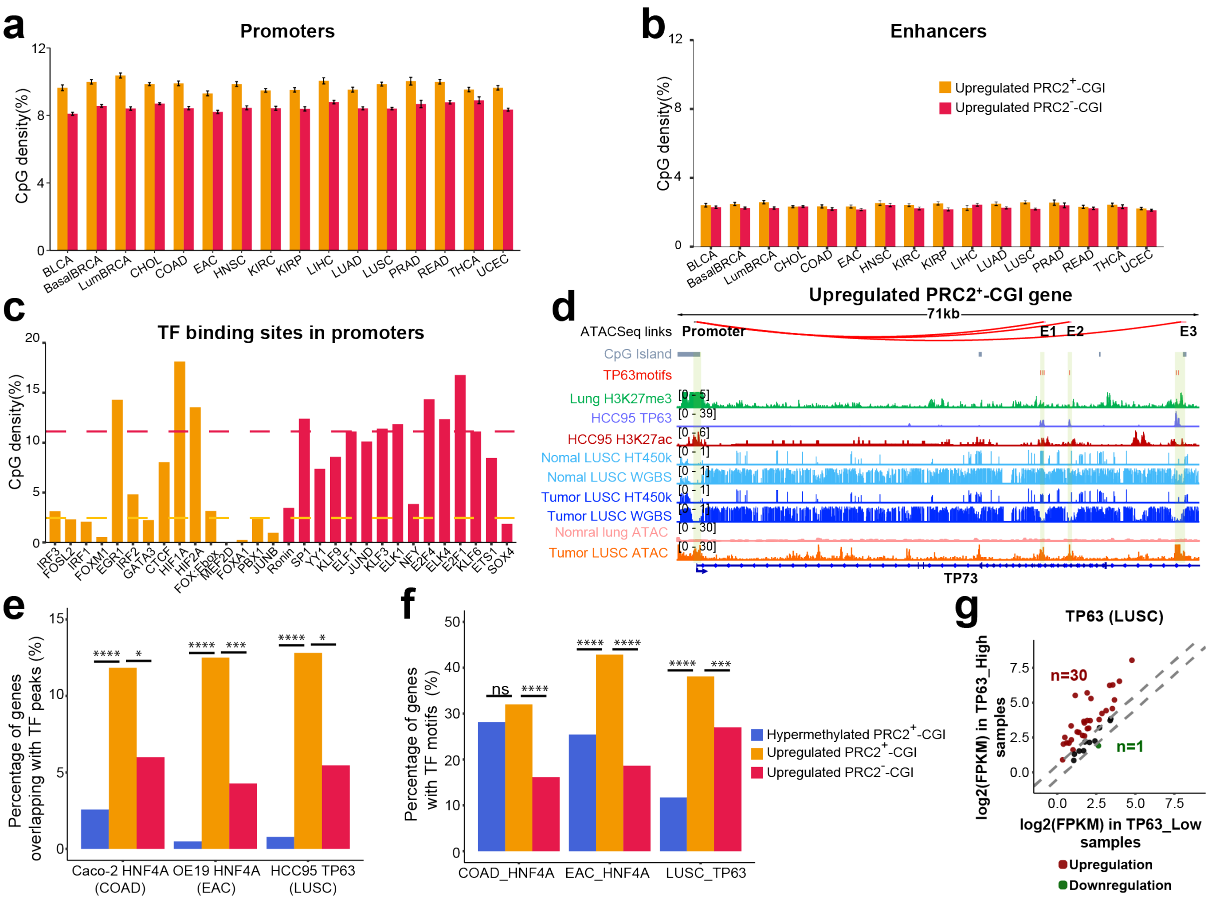

Supplementary Figure 11. Upregulated PRC2$^+$-CGI genes are linked to distal enhancers targeted by specific transcription factor binding sites (TFBSs). (a-b) The CpG density of promoters **(a)** and enhancers **(b)** in upregulated CGI gene classes. The error bars show the standard errors. Data are presented as mean values ± standard errors. The promoter/enhancers numbers (n) of each class in each cancer type are listed in **Supplementary Table 2**. **(c)** The CpG density of the top15 enriched TF binding sites in promoters. **(d)** TP73 enhancers (E1, E2, E3) predicted with TP63 motifs are occupied by TP63 in LUSC cells. TP63 ChIP-seq are from GSE66992. **(e)** TF ChIP-Seq of HNF4A/TP63 binding overlapping three CGI gene groups. For better comparison, the results of **Fig. 5i** are also incorporated. n=106 independent tumor samples in each group. **(f)** Percentage of genes with TF motifs. Motifs of gene associated enhancers were obtained using HOMER scanMotifGenomeWide.pl. The linked enhancers are from “enhancer-to-gene links" defined by the TCGA consortium. P values in panel (**e**) and (**f**) were determined by a two-sided Fisher’s exact test. p<0.0001, ****; p<0.001, ***; p<0.01, **; p<0.05, *. The exact P values are shown in **Supplementary Data 4. (g)** Expression differences between TCGA TP63-high and TP63-low LUSC tumors for the TP63 target genes having enhancers overlapped by TP63 in LUSC cells (from panel **e**). High and low tumors were those in the upper and lower quintile of TP63 expression. The cutoff for coloring is absolute fold change>=1.5.

Supplementary Tables

Supplementary Table 1. Normal and tumor sample distribution across cancer types

Cancer[a]	Patients with expression		Patients with methylation		Study in this work?
	Nonmalignant	Tumor	Nonmalignant	Tumor	
BLCA	19	408	21	412	Yes
BasalBRCA	101	190	75	136	Yes
LumBRCA	101	779	75	567	Yes
CHOL	9	36	9	36	Yes
COAD	41	456	39	295	Yes
EAC	8	77	11	86	Yes
HNSC	44	500	50	528	Yes
KIRC	72	530	160	319	Yes
KIRP	32	288	45	275	Yes
LIHC	50	371	50	377	Yes
LUAD	59	513	32	458	Yes
LUSC	49	501	42	370	Yes
PRAD	52	495	50	498	Yes
READ	10	166	7	98	Yes
THCA	58	502	56	507	Yes
UCEC	35	543	46	431	Yes
ESCC	1	76	1	90	No
ACC	0	79	0	80	No
CESC	3	304	3	307	No
DLBC	0	48	0	48	No
GBM	5	161	2	141	No
KICH	24	65	0	66	No
LAML	0	151	0	194	No
LGG	0	511	0	516	No
MESO	0	86	0	87	No
OV	0	376	0	10	No
PAAD	4	177	10	184	No
PCPG	3	179	3	179	No
SARC	2	259	4	261	No
SKCM	1	468	2	470	No
STAD	32	375	2	395	No
TGCT	0	150	0	150	No
THYM	2	119	2	124	No
UCS	0	56	0	57	No
UVM	0	80	0	80	No

[a]BLCA, bladder cancer; BasalBRCA, basal breast cancer; LumBRCA, luminal breast cancer; CHOL, cholangiocarcinoma; COAD, colon adenocarcinoma; EAC, esophageal adenocarcinoma; HNSC, head and neck squamous cell carcinoma; KIRC, kidney renal clear cell carcinoma; KIRP, kidney renal papillary cell carcinoma; LIHC, liver cancer; LUAD, lung adenocarcinoma; LUSC, lung squamous cell carcinoma; PRAD, prostate adenocarcinoma; READ, rectum adenocarcinoma; THCA, thyroid carcinoma; UCEC, uterine corpus endometrial carcinoma; ESCC, esophageal squamous cell carcinoma; ACC, adrenocortical carcinoma;

CESC, cervical squamous cell carcinoma and endocervical adenocarcinoma; DLBC, lymphoid neoplasm diffuse large B-cell lymphoma; GBM, glioblastoma multiforme; KICH, kidney chromophobe; LAML, acute myeloid leukemia; LGG, brain lower grade glioma; MESO, mesothelioma; OV, ovarian serous cystadenocarcinoma; PAAD, pancreatic adenocarcinoma; PCPG, pheochromocytoma and paraganglioma; SARC, sarcoma; SKCM, skin cutaneous melanoma; STAD, stomach adenocarcinoma; TGCT, testicular germ cell tumors; THYM, thymoma; UCS, uterine carcinosarcoma; UVM, uveal melanoma.

Supplementary Table 2. Gene and promoter distribution of different CGI categories across cancer types

Cancer	Hypermethylated PRC2$^+$-CGI		Upregulated PRC2$^+$-CGI			Upregulated PRC2$^-$-CGI		
	Genes	Promoters	Genes	Promoters	linked enhancers	Genes	Promoters	linked enhancers
BLCA	572	823	159	275	491	450	807	861
BasalBRCA	395	552	252	426	729	613	1,117	1,071
LumBRCA	887	1,325	219	416	732	415	766	892
CHOL	1,078	1,587	549	1,043	1,631	1,168	2,259	2,099
COAD	970	1,509	228	417	675	434	769	678
EAC	405	595	224	413	699	397	717	862
HNSC	779	1,132	197	361	462	285	536	548
KIRC	300	398	358	709	927	343	587	541
KIRP	152	173	251	487	692	333	561	577
LIHC	844	1,177	123	218	312	422	831	760
LUAD	880	1,344	218	384	596	494	898	1,037
LUSC	511	722	320	587	1,028	769	1,370	1,333
PRAD	534	725	98	179	278	126	208	261
READ	631	943	225	415	658	479	851	730
THCA	12	14	245	465	726	135	232	385
UCEC	545	790	268	487	1,012	541	958	1,228
Total	2,274	4,620	1,543	2,891	4,937	2,521	3,668	4,661

Supplementary Table 3. siRNA and primers used in this study

	Sense (5′-3′)	Antisense (5′-3′)
**siRNAs**		
siMATN3-1	ACAUUUUGGAGAAGUUGAAdtdt	UUCAACUUCUCCAAAAUGUdtdt
siMATN3-2	GUGAGUGCUAUGAAGGUUAdtdt	UAACCUUCAUAGCACUCACdtdt
siSERPINE2-1	UGGAGUUGGUAAAAUAUUAdtdt	UAAUAUUUUACCAACUCCAdtdt
siSERPINE2-2	CCAAGAAGAAUAAAGACAUdtdt	AUGUCUUUAUUCUUCUUGGdtdt
siDKK1	AUGUGUGUCUUCUGAUCAAdtdt	UUGAUCAGAAGACACACAUdtdt
siHNF4A	CCAAGUACAUCCCAGCUUUdtdt	AAAGCUGGGAUGUACUUGGdtdt
Scramble	UUCUCCGAACGUGUCACGUdtdt	ACGUGACACGUUCGGAGAAdtdt
**Primers for RT-PCR**		
APLP1	TCCCCTGAGAAAGAGAAGATGAACC	TCTGAATCTCCGATGAGTGGAAAGG
CRLF1	CACATCCCCAAGGACCTGGCTCTCT	ACATCGTCCACCACCTTCCAGTCCA
MATN3	AGCAGACCCTTGGACCTGGTGTTTA	GTGCCTGTTGACAAGGGTGTGATTC
SERPINE2	AAGATGTGTTCCAGTGTGAGGTCCG	AACCGTGATTTCCACAGACCCTTGA
DKK1	ATTCCAACGCTATCAAGAACCTGCC	CGGCTGGTAGTTGTCAATGGTCTGG
Slug	ATGCATATTCGGACCCACACATTAC	AGATTTGACCTGTCTGCAAATGCTC
Vimentin	GCTGAATGACCGCTTCGCCAACT	GCTCCCGCATCTCCTCCTCGTA
Fibronectin	TGAAAGACCAGCAGAGGCATAAG	CTCATCTCCAACGGCATAATGG
N-Cadherin	ACCAGGTTTGGAATGGGACAG	ATGTTGGGTGAAGGGGTGCTTG
GAPDH	GTCTCCTCTGACTTCAACAGCG	ACCACCCTGTTGCTGTAGCCAA
HNF4A	CATCAGAAGGCACCAACCTCAACGC	TCATTCTGGACGGCTTCCTTCTTCA
CDH1	TGGATAGAGAACGCATTGCCACATA	GTGTAAGCGATGGCGGCATTGTA
JUP	CGTAGGGTCTTTCTTGGGATAGTGT	TGGAGTTCAGTGAGAAAATCAGACC
KRT8	ATGGGAGGCATCACCGCAGTTAC	AGGCAAACTTGTTGTTGAGGGTCTT
KRT14	GGAACAAGATTCTCACAGCCACAGT	TCTCCACATTGACATCTCCACCCAC
β-Actin	AGCGAGCATCCCCCAAAGTT	GGGCACGAAGGCTCATCATT
**Primers for ChIP-PCR**		
Negative control-P	ACCCGTCTTCGACAGGACT	GGAACGGAAGACGAGAACAG
EFNA2-P	GTGTCAGCCTACCAGGAGGAGAAAT	GAGTAAGTCCCTACACCTCAGCCCT
CLDN1-P	CGCAGGACATCCACAGCCCCTCGTA	CTGTTGGGCTTCATTCTCGCCTTCC
SLC15A1-P	TTAGACGGTGTTTCTGCCGCCCCTG	CCGTGGGGCACTGTAATCCTCGTAT
IRAK2-P	TAGTGCCGATGGAAGTGGGGTCAGA	GAGAATCGGAACTGCGAGGGGAAAC
P2RY2-P	GAGCAGGTATCTCCCAGTAGGTCCC	TACCAGAGGTTTTCAGAGACGCCAA
CXCL1-P	CAGCCTATCTCCCAGCACCTTGTTA	CAGTGTGTGGGGCTGGGAACGGAGA

Supplementary references

1. Xie, W. *et al.* Epigenomic analysis of multilineage differentiation of human embryonic stem cells. *Cell* **153**, 1134–1148 (2013).

2. Miao, D. *et al.* Genomic correlates of response to immune checkpoint therapies in clear cell renal cell carcinoma. *Science* **359**, 801–806 (2018).
