# Supplementary material for: A pan-cancer analysis of CpG Island gene regulation reveals extensive plasticity within Polycomb target genes
Source: Nat Commun. 2021 Apr 30;12:2485. doi: 10.1038/s41467-021-22720-0 (PMC8087678; doi:10.1038/s41467-021-22720-0)
Supplement: Supplementary file 2 — Description of Additional Supplementary Files [file 41467_2021_22720_MOESM2_ESM.pdf]

Description of Additional Supplementary Files

File Name: Supplementary Data 1
Description: Hypermethylated $PRC2^{+}$-CGI genes across different cancer types

File Name: Supplementary Data 2
Description: Upregulated $PRC2^{+}$-CGI genes across different cancer types

File Name: Supplementary Data 3
Description: Upregulated $PRC2^{-}$-CGI genes across different cancer types

File Name: Supplementary Data 4
Description: The exact p values of all statistical tests
