# Supplementary material for: A pan-cancer analysis of CpG Island gene regulation reveals extensive plasticity within Polycomb target genes
Source: Nat Commun. 2021 Apr 30;12:2485. doi: 10.1038/s41467-021-22720-0 (PMC8087678; doi:10.1038/s41467-021-22720-0)
Supplement: Supplementary file 7 — Reporting Summary [file 41467_2021_22720_MOESM7_ESM.pdf]

nature research

Corresponding author(s): De-Chen Lin, Benjamin P. Berman

Last updated by author(s): Feb 28, 2021

Reporting Summary

Nature Research wishes to improve the reproducibility of the work that we publish. This form provides structure for consistency and transparency in reporting. For further information on Nature Research policies, see our Editorial Policies and the Editorial Policy Checklist.

Statistics

For all statistical analyses, confirm that the following items are present in the figure legend, table legend, main text, or Methods section.

n/a	Confirmed	
☐	☒	The exact sample size (n) for each experimental group/condition, given as a discrete number and unit of measurement
☐	☒	A statement on whether measurements were taken from distinct samples or whether the same sample was measured repeatedly
☐	☒	The statistical test(s) used AND whether they are one- or two-sided<br>*Only common tests should be described solely by name; describe more complex techniques in the Methods section.*
☒	☐	A description of all covariates tested
☐	☒	A description of any assumptions or corrections, such as tests of normality and adjustment for multiple comparisons
☐	☒	A full description of the statistical parameters including central tendency (e.g. means) or other basic estimates (e.g. regression coefficient) AND variation (e.g. standard deviation) or associated estimates of uncertainty (e.g. confidence intervals)
☐	☒	For null hypothesis testing, the test statistic (e.g. F, t, r) with confidence intervals, effect sizes, degrees of freedom and P value noted<br>*Give P values as exact values whenever suitable.*
☒	☐	For Bayesian analysis, information on the choice of priors and Markov chain Monte Carlo settings
☒	☐	For hierarchical and complex designs, identification of the appropriate level for tests and full reporting of outcomes
☐	☒	Estimates of effect sizes (e.g. Cohen's d, Pearson's r), indicating how they were calculated

Our web collection on statistics for biologists contains articles on many of the points above.

Software and code

Policy information about availability of computer code

Data collection	The TCGAbiolinks package(v 2.13.6) was used to download the sample information, mRNA expression (RNA-Seq level 3 data) and DNA methylation (Illumina HumanMethylation450 array) data of 33 types of cancers (n=10,528) from the TCGA project (GDC v16.0). Annotation of CpG Island (CGI) regions was downloaded from UCSC website (http://hgdownload.soe.ucsc.edu/goldenPath/hg38/database/).
Data analysis	ChIP-Seq data were analyzed by Bowtie (v 1.2.2, for reads length shorter than 51bp), Bowtie2 (v2.3.4.3, for reads longer than 51bp), SAMtools (v 1.3.1), Picard MarkDuplicates (v 1.136), betools (v 2.27.1), MACS2 (v 2.1.2) and UCSC bedGraphToBigWig tools (v 4).<br>ATAC-Seq data analysis were performed using Bowtie2, SAMtools, MACS2.<br>RNA-Seq data were analyzed by HISAT2(v 2.0.4), htseq-count (v 0.11.2) and DESeq2 package (v 1.22.2).<br>PCA analysis was performed using R prcomp function and point plots are generated by ggplot2 package (v 3.1.0).<br>Motif analysis were performed using HOMER findMotifsGenome.pl script (v 4.9.1) .

For manuscripts utilizing custom algorithms or software that are central to the research but not yet described in published literature, software must be made available to editors and reviewers. We strongly encourage code deposition in a community repository (e.g. GitHub). See the Nature Research guidelines for submitting code & software for further information.

Data

Policy information about availability of data

All manuscripts must include a data availability statement. This statement should provide the following information, where applicable:

- Accession codes, unique identifiers, or web links for publicly available datasets
- A list of figures that have associated raw data
- A description of any restrictions on data availability

ATAC-Seq data of TCGA samples and pan-cancer “enhancer-to-gene” links were obtained from Corces et al. 2018 (DOI: 10.1126/science.aav1898). Other datasets

were collected: H3K27ac ChIP-Seq in nonmalignant colonic crypts and primary colon cancer cells (GSE77737), H3K27ac ChIP-Seq in nonmalignant and tumor samples of kidney renal clear cell carcinoma (KIRC) from GSE86095, HNF4A ChIP-Seq in OE19 (E-MTAB-6858) and Caco-2 (GSE23436) cell lines, TP63 ChIP-Seq in HCC95 cell line (GSE46837), SP1 and JUND ChIP-Seq in HCT116 and A549 cell lines (ENCODE), H3K27ac ChIP-Seq in OE19 (GSE132686), HCC95 (GSE66992), HCT116 (ENCODE), Caco-2 (GSE96069) and A549 (ENCODE) cell lines. ATAC-Seq of nonmalignant esophageal epithelium, EAC tissues, normal esophageal cells (HET1A) and OE19 tumor cells are available in ArrayExpress database under accession code E-MTAB-5169 and E-MTAB-6931; We also collected ATAC-Seq datasets of nonmalignant lung tissue, lung adenocarcinoma and lung squamous cell carcinoma downloaded from NSCLC ATAC-seq project at. https://pms.cd120.com/download.html. RNA-Seq of HNF4A knockdown are available in ArrayExpress database under accession code E-MTAB-6756. RNA-Seq datasets from pre-treatment tumors with anti-PD-1 monotherapy in KIRC were obtained from Miao et al. 2018 (DOI: 10.1126/science.aan5951). We also retrieved the mRNA expression data of basal breast cancer cell lines from the Cancer Cell Line Encyclopedia (CCLE). Annotation of CpG Island (CGI) regions was downloaded from UCSC website (http://hgdownload.soe.ucsc.edu/goldenPath/hg38/database/).The sample information, mRNA expression (RNA-Seq level 3 data) and DNA methylation (Illumina HumanMethylation450 array) data of 33 types of cancers (n=10,528) are available in TCGA project (GDC v16.0). The mRNA expression data of basal breast cancer cell lines is available in CCLE.

Field-specific reporting

Please select the one below that is the best fit for your research. If you are not sure, read the appropriate sections before making your selection.

☒ Life sciences ☐ Behavioural & social sciences ☐ Ecological, evolutionary & environmental sciences

For a reference copy of the document with all sections, see nature.com/documents/nr-reporting-summary-flat.pdf

Life sciences study design

All studies must disclose on these points even when the disclosure is negative.

Sample size	We didn't perform computation to pre-determine the sample size for the perturbation experiments. We justified the sample size based on published papers, our extensive published experience with similar experiments, and those generally employed in the field. The results obtained suggest the chosen sample size is appropriate because either clear distinctions are observed or the results have reached statistical significance.
Data exclusions	No data was excluded in the perturbation experiments.
Replication	All perturbation experiments were performed in duplicate or triplicate biological experiments. Each biological replicate contained 2-3 technical replicates. All replications were showing consistent results.
Randomization	No method of randomization was used as we utilized all available data to maximize the sample size and none of the experiments involved blinded or unblinded allocation of samples to test groups.
Blinding	No blinding was required in our study as no group allocation was involved in this study.

Reporting for specific materials, systems and methods

We require information from authors about some types of materials, experimental systems and methods used in many studies. Here, indicate whether each material, system or method listed is relevant to your study. If you are not sure if a list item applies to your research, read the appropriate section before selecting a response.

Materials & experimental systems

n/a	Involved in the study	
☐	☒	Antibodies
☐	☒	Eukaryotic cell lines
☒	☐	Palaeontology and archaeology
☒	☐	Animals and other organisms
☒	☐	Human research participants
☒	☐	Clinical data
☒	☐	Dual use research of concern

Methods

n/a	Involved in the study	
☒	☐	ChIP-seq
☒	☐	Flow cytometry
☒	☐	MRI-based neuroimaging

Antibodies

Antibodies used	H3K27Me3 (#ab6002, Abcam)
Validation	H3K27Me3 antibodies are validated for ChIP-qPCR in numerous publications(PMID: 25101834, 32428001).

Eukaryotic cell lines

Policy information about cell lines

Cell line source(s)	HS578T cells (ATCC #HTB-126), MDAMB436 cells (ATCC #HTB-130) and OE19 cells(from Sigma-Aldrich)
Authentication	HS578T and MDAMB436 were authenticated by ATCC. All ATCC cell lines undergo authentication tests by Short Tandem Repeat (STR) analysis for human cell lines. OE19 were recently authenticated by STR analysis.
Mycoplasma contamination	All cell lines used in-house tested negative for mycoplasma.
Commonly misidentified lines (See ICLAC register)	No cell lines from the ICLAC register were used.
